# Network meta-analysis of episiotomy approaches: comparing routine, restrictive, and non-episiotomy strategies and their effects on maternal and neonatal outcomes in primiparous patients

**DOI:** 10.1186/s12978-025-02132-9

**Published:** 2025-09-16

**Authors:** Mahmoud Shaaban Abdelgalil, Basma Ehab Amer, Elsayed Eldesouky, Moaz Yasser Darwish, Elsayed Farag, Attia Mohamed, Mohammed Ali, Alaa Abdelsalam, Zeinab Yousef Hashem, Esraa Elsayed, Reem M. Elsapagh, Marwa M. Badawy, Mohamed Abd-ElGawad, Almandouh H. Bosilah

**Affiliations:** 1 Faculty of Medicine, Ain-shams University, 359 Abd Allah Nadim Street, Cairo, Egypt; 2 Faculty of Medicine, Benha University, Benha, Egypt; 3 Department of Obstetrics and Gynecology, Faculty of Medicine, Al-Azhar University, Cairo, Egypt; 4 Faculty of Medicine, Fayoum University, Fayoum, Egypt; 5 Faculty of Medicine, Al-Azhar University, Cairo, Egypt; 6 Faculty of Medicine, Zagazig University, Zagazig, Egypt; 7 Faculty of Pharmacy, Cairo University, Cairo, Egypt; 8 Faculty of Medicine, October 6 University, Giza, Egypt; 9 Department of Obstetrics and Gynecology, Faculty of Medicine, Damietta University, Damietta, Egypt

**Keywords:** Episiotomy, Perineal trauma, Post-partum morbidities

## Abstract

**Background:**

Episiotomy, although occasionally required during labor, can result in postpartum discomfort, perineal trauma, and delayed healing. This study offers a comprehensive comparison of the safety and effectiveness of routine, restrictive, and non-episiotomy approaches on maternal and neonatal outcomes.

**Methods:**

We systematically searched PubMed, Web of Science, Scopus, and Cochrane. We included randomized clinical trials (RCTs), comparing routine, restrictive, and non-episiotomy approaches in primiparous pregnant women. We excluded non-randomized trials, conference abstracts, and observational studies. The Cochrane ROB tool 2 was employed to appraise the selected RCTs. We conducted our analysis using the “netmeta” package of RStudio software (v.4.3.0). Dichotomous outcomes were pooled as risk ratios (RR), while continuous outcomes were pooled as the mean differences (MD). We used the random effect model for all outcomes.

**Results:**

Sixteen studies, involving 10,738 patients, were included in the network meta-analysis. The findings revealed that the non-episiotomy group had a significantly higher risk of experiencing 1st-degree perineal tear compared to the routine episiotomy group (RR = 4.69, 95% CI [2.04; 10.74], *P* > 0.01). Similarly, the restrictive episiotomy group showed a significantly higher risk compared to the routine group (RR = 3.88, 95% CI [1.63; 9.26], *P* > 0.01). However, there were no significant differences observed between the routine, restrictive, and non-episiotomy groups regarding the duration of the 2nd stage of labor, postpartum perineal pain, and the occurrence of 2nd-, 3rd-, and 4th-degree perineal tears.

**Conclusion:**

In conclusion, non-episiotomy approaches prove superior, with lower risks of complications compared to routine episiotomy. While restrictive episiotomy performs better than routine, it falls short of non-episiotomy methods. Therefore, non-episiotomy strategies are preferred, followed by restrictive episiotomy, with routine episiotomy being the least favorable. However, individual patient factors should guide treatment decisions, and further research is necessary to refine clinical practices.

**Supplementary Information:**

The online version contains supplementary material available at 10.1186/s12978-025-02132-9.

## Introduction

The practice of episiotomy—a surgical incision to widen the birth canal—dates back to 1742 when Sir Fielding Ould first introduced it to minimize maternal trauma [1]. Despite its initial adoption without robust scientific evidence, episiotomy gained widespread acceptance in the 20th century following recommendations from leading obstetricians like Gabbe and DeLee [2, 3]. Consequently, its routine use led to a sharp rise in episiotomy rates, particularly in hospital-based deliveries, increasing from 5% in 1900 to nearly 80% by 1950 [4]. Currently, episiotomies are performed in one in three vaginal deliveries, with rates 8.8 times higher in primiparous than multiparous women [5]. Despite the WHO’s 1996 recommendation to limit episiotomies to 10% of vaginal deliveries, global rates vary significantly, ranging from 8% in the Netherlands to 25% in the US, with Taiwan reaching 100% [6]. However, rising complications have prompted a shift toward a more conservative approach [7].

Historically, episiotomy was believed to prevent severe perineal tears, neonatal complications, and long-term maternal sequelae [8]. However, emerging evidence has challenged these assumptions. The landmark 1983 review by Thacker and Banta found insufficient evidence supporting routine episiotomy and instead highlighted its associated risks, including perineal pain, hematoma, infection, dyspareunia, and healing complications [9]. As a result, professional organizations, including the American Congress of Obstetricians and Gynecologists, have shifted away from routine episiotomy in favor of a restrictive approach, reserving the procedure for specific clinical indications [10, 11].

Despite this shift, the effectiveness of different episiotomy strategies remains a subject of debate. While some studies suggest that restrictive episiotomy reduces complications [1, 12]. others, including a meta-analysis by Pereira et al., found no significant differences between restrictive and non-episiotomy approaches [13–15]. Given this ongoing controversy, a more sophisticated analytical approach is required to compare these strategies comprehensively.

To date, no study has employed network meta-analysis to evaluate episiotomy strategies. Network meta-analysis enables simultaneous comparison of multiple interventions, integrating both direct and indirect evidence to improve statistical power and precision [16–18].

This study aims to address this gap by conducting a network meta-analysis to assess the effects of routine, restrictive, and non-episiotomy strategies on maternal and neonatal outcomes in primiparous patients. By synthesizing available evidence, our findings will provide a clearer ranking of episiotomy strategies to inform clinical decision-making.

## Methods

We performed our network meta-analysis (NMA) according to the Preferred Reporting Items for Systematic Reviews and Meta-Analyses (PRISMA) guidelines [19] and the Cochrane handbook of systematic intervention [20].

### Literature search and data collection

We searched PubMed, Web of Science, Scopus, and Cochrane library from inception until December 15, 2023. We used the following terms: episiotomy, episiotomies, perineotomy, and non-episiotomy. The detailed search strategy for each database is provided in Supplementary file Table 1.

We searched for any published results of ongoing trials. Duplications were removed using Endnote software version 21.2 for Windows, and all search received criteria assessed the eligibility through a title, abstract, and full-text screening, we included the papers that matched our criteria. We searched manually for other included studies for other related documents. We updated our search again on March 25, 2024, to avoid missing any recently published studies.

### Eligibility criteria and study selection

We included all relevant randomized clinical trials (RCTs), which compared routine, restrictive episiotomy, and non-episiotomy (spontaneous tear) approaches in primiparous pregnant women. Exclusion criteria for this meta-analysis encompass non-randomized trials, observational studies, and those not explicitly comparing routine, restrictive, and non-episiotomy approaches in primiparous women. Additionally, studies lacking sufficient data, not published in English were excluded. Non-peer-reviewed sources and conference abstracts were also excluded to prioritize high-quality evidence and ensure the robustness of the analysis.

### Methodological quality assessment

We conducted a rigorous quality assessment using the Cochrane Risk of Bias 2 tool [21] to evaluate the methodological quality of the included studies. This tool enabled a comprehensive evaluation across key domains such as randomization, deviations from intended interventions, missing outcome data, outcome measurement, and selection of reported results. The systematic categorization of each study into low, some concerns, or high risk of bias within these domains enhances the transparency and credibility of our NMA.

### Data extraction

In our data retrieval process, we organized essential information into Excel sheets, encompassing various key elements. The first sheet, labeled “Summary,” includes details, such as study ID, site, country, National Clinical Trial (NCT) identifier, inclusion criteria, study intervention, sample size, and conclusion. The second sheet, titled “Baseline characteristics,” comprises study ID, mother’s age, intervention and control details, gestational age at delivery, birth weight, and BMI. Finally, the third sheet captures the study outcomes. Our primary outcomes included the duration of 2nd stage of labor, post-partum perineal pain, 1st, 2nd, 3rd, and 4th degrees perineal tear. However, our secondary outcomes were divided into maternal outcomes, such as post-partum blood loss > 500 ml, wound hematoma, wound infection, urinary incontinence, and anal incontinence, and neonatal outcomes, such as 1st minute and 5-minute Apgar score < 7. Two independent authors were responsible for data extraction and a third author was consulted to resolve any discrepancies.

### Outcomes definition

The maternal outcomes assessed in this study encompass a range of health indicators. Wound infection is defined by the presence of infectious microorganisms at the episiotomy site, clinically evident through signs such as redness and swelling [22]. Postpartum blood loss exceeding 500 milliliters signifies excessive hemorrhage during the postpartum period [23]. The duration of the second stage of labor measures the time from complete cervical dilation to the baby’s delivery, offering insights into the labor progress [24]. Wound hematoma refers to blood accumulation at the episiotomy site, causing swelling. Postpartum perineal pain reflects discomfort experienced by mothers and can be self-reported or clinically assessed [25]. Anal incontinence signifies the inability to control bowel movements, while urinary incontinence refers to the involuntary loss of urine [26]. Additionally, the classification of perineal tears from 1st to 4th degree provides a comprehensive evaluation of perineal trauma. Neonatal outcomes, including 1st-minute and 5-minute Apgar scores below 7, serve as indicators of potential neonatal distress shortly after birth [26].

### Data synthesis

The NMAs were executed through Frequency analysis in the R program (v.4.3.0) with the “netmeta” package [27] in RStudio, adhering to our study protocol. For dichotomous outcomes, risk ratios (RR) were computed, and mean differences (MD) were aggregated for continuous outcomes. Each NMA generated three visual representations tailored to our study’s specific focus: (A) a network plot illustrating patient distribution in each group with lines delineating direct comparisons among routine, restrictive, and non-episiotomy (spontaneous tear) approaches, (B) a forest plot depicting the effect estimate of each approach relative to the reference group, and (C) a league table organizing approaches based on their superiority. Statistical significance was determined at *P* values < 0.05. Heterogeneity was assessed using the I² test, with significance declared at *P* value < 0.1. The random-effect model was employed for all outcomes because we suspected the presence of heterogeneity among the included studies. The order of ranking was depicted using surface under the cumulative ranking curves (SUCRA), where a higher SUCRA value indicated a greater probability of the intervention being in the top-ranking position [28].

## Results

### Literature search results

**Fig. 1:**
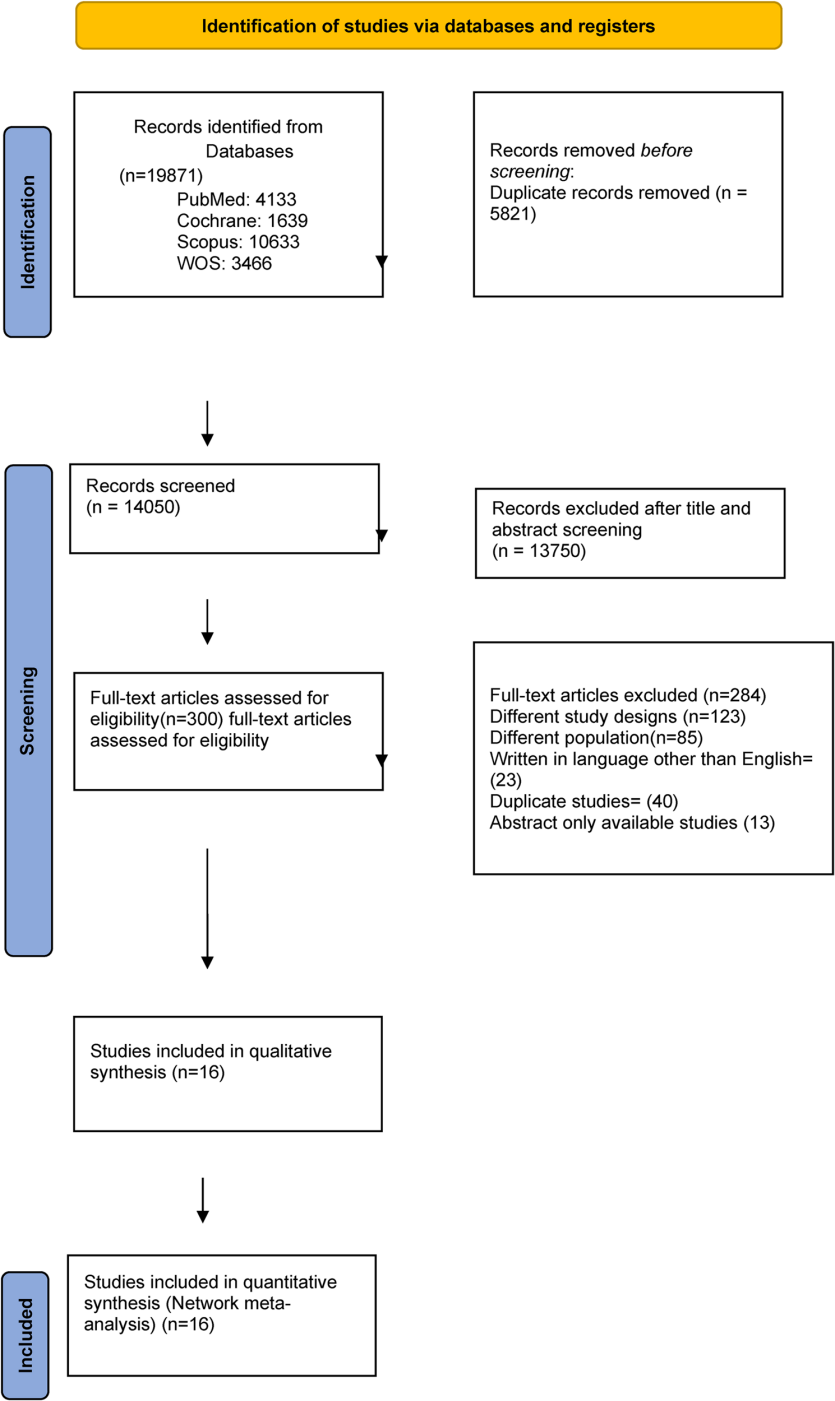
PRISMA flow diagram for included studies

Applying our search strategy to different databases retrieved 19,871 records. After screening the titles and abstracts of these records, we had 300 articles for full-text screening. Sixteen eligible articles [4, 14, 29–42] were incorporated in our network meta-analysis (Fig. 1).

### Characteristics of the included studies

Nine studies compared routine with restrictive episiotomy [4, 29–31, 35, 37, 38, 41, 42], whereas three studies compared routine episiotomy with no episiotomy [32, 39, 40], and four studies compared restrictive episiotomy with no episiotomy [14, 33, 34, 36]. The mean age of mothers ranged across studies from 19.7 in Rodriguez et al. [31] to 28.5 in Sajid et al. [40]. The mean gestational age at delivery ranged from 37.9 weeks to 40.5 weeks. The mean Birth weight ranged across studies from 2899 gram in Rodriguez et al. [31] to 3589 gram in Murphy et al. [35]. The included studies span from 1988 to 2021 and were conducted across eleven countries: Brazil (1), Iran (1), Sweden (1), Israel (1), Pakistan (2), the United Kingdom (4), Malaysia (1), Colombia (1), Thailand (4), Saudi Arabia (1), and Argentina (1) (Supplementary file Table 2 and Table 3).

All 16 studies showed a low risk of bias regarding missing outcome data, except for two studies [4, 40]. Similarly, in terms of outcome measurement, all studies were at low risk, except for four studies [4, 33, 36, 40]. Ten studies demonstrated low risk of bias for the randomization process [4, 14, 29, 33–37, 41, 42], while seven studies were at low risk for deviation from the intended intervention [14, 29, 30, 34, 35, 41, 43]. Most studies had an unclear risk of bias in terms of selection of reported results, except for four studies [14, 33, 34, 36] which were low risk (Supplementary file Fig. 1).

### Primary outcomes

#### Duration of 2nd stage of labor

##### Pairwise meta-analysis result

**Fig. 2:**
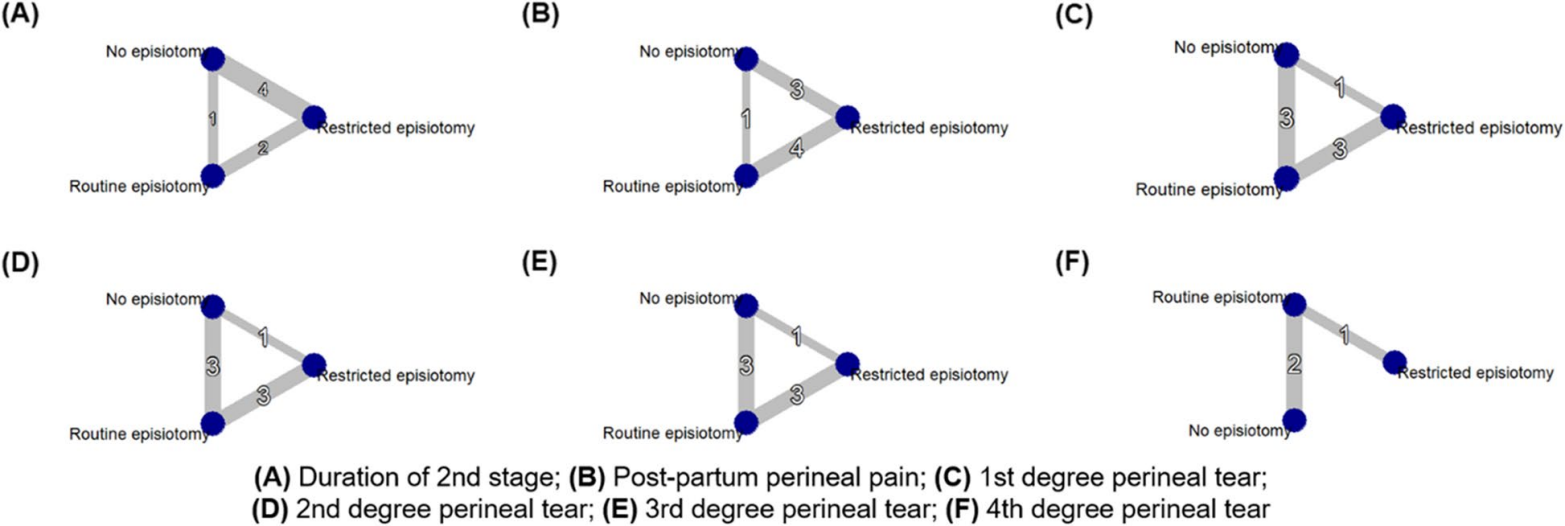
Network graphs for primary outcomes

**Fig. 3:**
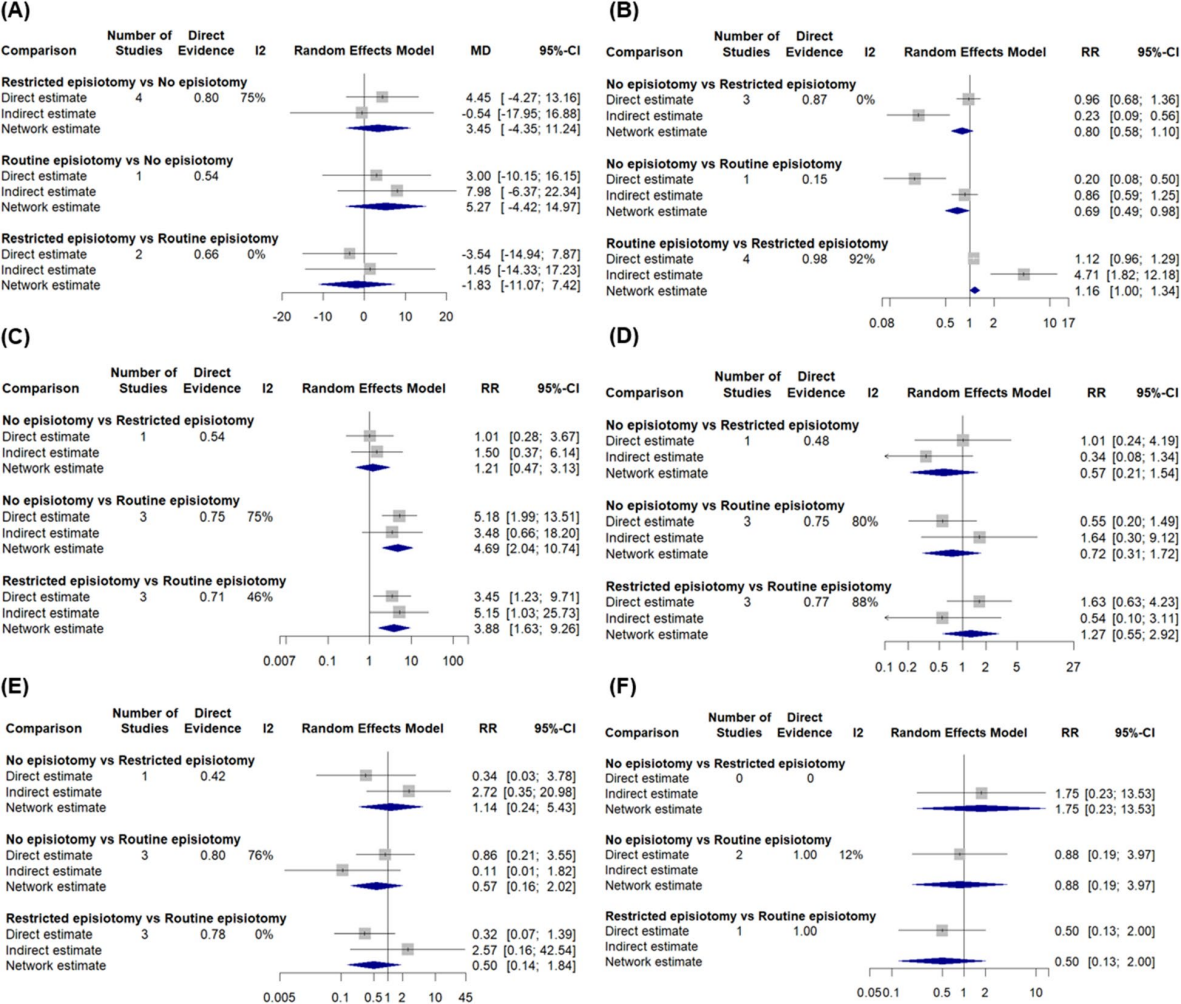
Pairwise meta-analysis for primary outcomes **A** Duration of 2nd stage; **B** Post-partum perineal pain; **C** 1st degree perineal tear; **D** 2nd degree perineal tear; **E** 3rd degree perineal tear; **F** 4th degree perineal tear

Four studies [14, 33, 34, 36] comparing between the restrictive group and the non-episiotomy group showed a non-significant mean difference (MD = 4.45, 95% CI [−4.27, 13.16]). Additionally, one study [32] comparing between the routine group and the non-episiotomy group found a non-significant mean difference (MD = 3.00, 95% CI [−10.15, 16.15]). Furthermore, two studies [29, 37] comparing between the restrictive and the routine episiotomy group revealed a non-significant mean difference (MD = −3.54, 95% CI [−14.94, 7.87]) (Figs. 2A and 3A).

##### Network meta-analysis result and ranking

**Fig. 4:**
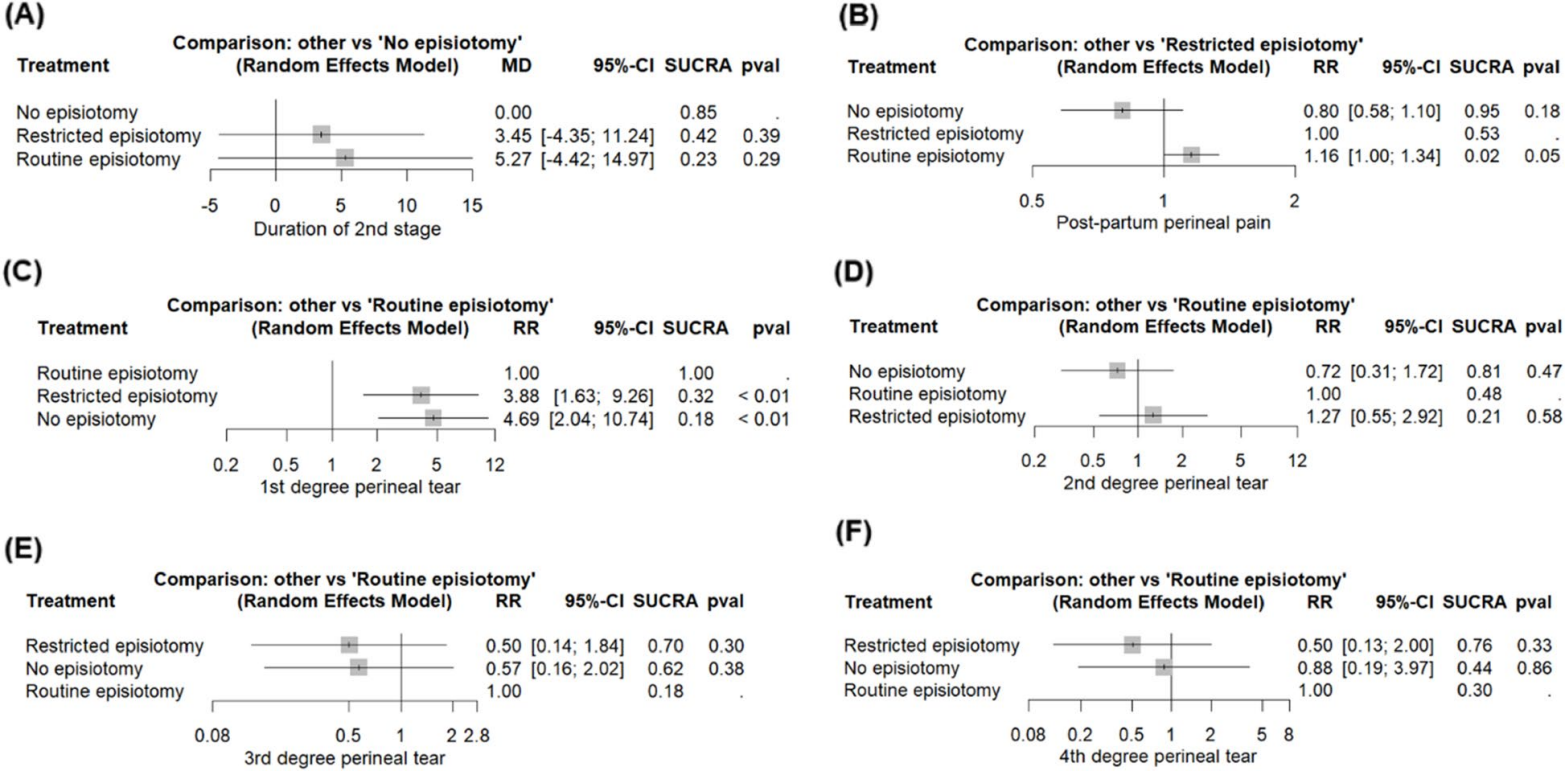
Forest plots of network meta-analysis for primary outcomes **A** Duration of 2nd stage; **B** Post-partum perineal pain; **C** 1st degree perineal tear; **D** 2nd degree perineal tear; **E** 3rd degree perineal tear; **F** 4th degree perineal tear

**Fig. 5:**
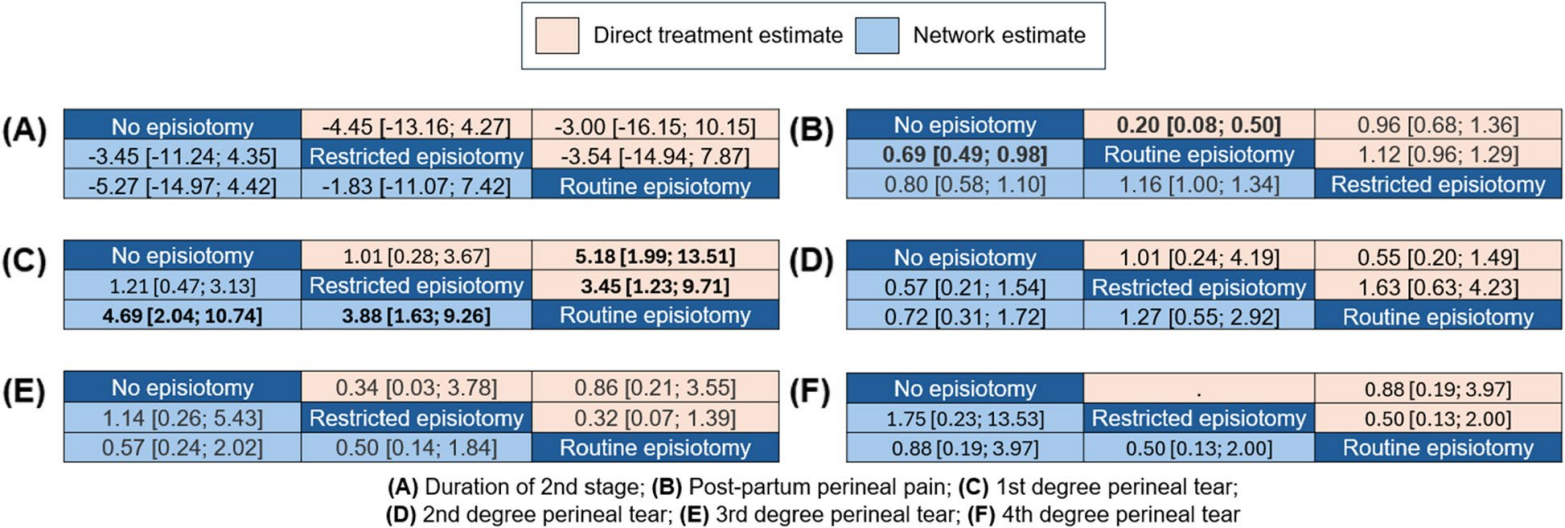
League tables for primary outcomes **A** Duration of 2nd stage; **B** Post-partum perineal pain; **C** 1st degree perineal tear; **D** 2nd degree perineal tear; **E** 3rd degree perineal tear; **F** 4th degree perineal tear

The non-episiotomy groups ranked first, followed by the restrictive episiotomy group and, subsequently, routine episiotomy group. The network meta-analysis demonstrated non-significant effects between the routine and non-episiotomy groups (MD = 5.27, 95% CI [−4.42, 14.97]), and the restrictive and non-episiotomy groups (MD = 3.45, 95% CI [−4.35, 11.24]). The pooled studies were heterogenous (I² = 59.8%, *P* = 0.03) (Figs. 4A and 5A).

#### Post-partum perineal pain

##### Pairwise meta-analysis result

In three studies [14, 34, 36] comparing between the non-episiotomy and restrictive groups, a non-significant risk reduction was observed (RR = 0.96, 95% CI [0.68; 1.36]). Conversely, in one study [32] comparing between the routine and the non-episiotomy groups, a significant decrease in risk was observed with the non-episiotomy group (RR = 0.20, 95% CI [0.08; 0.50]). Additionally, four studies [4, 29, 41, 42] comparing between the restrictive and routine episiotomy groups indicated a non-significant risk reduction (RR = 1.12, 95% CI [0.96; 1.29]) (Figs. 2B and 3B).

##### Network meta-analysis result and ranking

The non-episiotomy group ranked first, followed by the restrictive episiotomy group, and finally, routine episiotomy group. The routine episiotomy group exhibited a non-significant effect, compared to the restrictive episiotomy group (RR = 1.16, 95% CI [1.00; 1.34]). Similarly, the non-episiotomy group was comparable to the restrictive episiotomy group (RR = 0.80, 95% CI [0.58; 1.10]). The pooled studies were heterogenous (I² = 88%, *P* < 0.0001) (Figs. 4B and 5B).

#### 1st degree perineal tear

##### Pairwise meta-analysis result

In one study [34] comparing between the non-episiotomy and restrictive episiotomy groups, a non-significant increased risk was observed (RR = 1.01, 95% CI [0.28; 3.67]). Conversely, in three studies [29, 30, 37] comparing between the non-episiotomy and routine episiotomy groups, a significant increase in risk was observed with the non-episiotomy group (RR = 5.18, 95% CI [1.99; 13.51]). Finally, three studies [32, 39, 40] comparing between the restrictive and routine episiotomy groups showed a significant increase in risk was with the restrictive episiotomy group (RR = 3.45, 95% CI [1.23; 9.71]) (Figs. 2C and 3C).

##### Network meta-analysis result and ranking

Routine episiotomy group ranked first, followed by restrictive episiotomy group, and finally, the non-episiotomy group. Notably, non-episiotomy episiotomy group exhibited a significantly higher effect than the routine episiotomy group (RR = 4.69, 95% CI [2.04; 10.74], *P* > 0.01). Similarly, the restrictive episiotomy group showed a significantly higher effect than the routine group (RR = 3.88, 95% CI [1.63; 9.26], *P* > 0.01). The pooled studies were heterogenous (I² = 58.9%, *P* = 0.03) (Figs. 4C and 5C).

#### 2nd degree perineal tear

##### Pairwise meta-analysis result

In one study [34] comparing between the non-episiotomy and the restrictive episiotomy groups, a non-significant increase in risk was observed (RR = 1.01, 95% CI [0.24; 4.19]). Similarly, in three studies [29, 30, 37] comparing between the non-episiotomy and routine episiotomy groups, a non-significant risk reduction was found (RR = 0.55, 95% CI [0.20; 1.49]). Finally, three studies [32, 39, 40] comparing between the restrictive and routine episiotomy groups indicated a non-significant higher risk (RR = 1.63, 95% CI [0.63, 4.23]) (Figs. 2D and 3D).

##### Network meta-analysis result and ranking

The non-episiotomy group ranked first, followed by the routine episiotomy group, and finally the restrictive episiotomy group. The network meta-analysis revealed non-significant effects between the non-episiotomy versus routine episiotomy groups (RR = 0.72, 95% CI [0.31; 1.72]), and the restrictive episiotomy versus routine episiotomy groups (RR = 1.27, 95% CI [0.55; 2.92]). The pooled studies were heterogenous (I² = 81.6%, *P* < 0.0001) (Figs. 4D and 5D).

#### 3rd degree perineal tear

##### Pairwise meta-analysis result

In one study [34] comparing between the non-episiotomy and restrictive episiotomy groups, a non-significant risk reduction was observed (RR = 0.34, 95% CI [0.03; 3.78]). Similarly, in three studies [29–31] comparing between the non-episiotomy and routine episiotomy groups, a non-significant risk reduction was found (RR = 0.86, 95% CI [0.21; 3.55]). Finally, three studies [32, 39, 40] comparing between the restrictive and routine episiotomy groups indicated a non-significant risk reduction (RR = 0.32, 95% CI [0.07, 1.39]) (Figs. 2E and 3E).

##### Network meta-analysis result and ranking

The restrictive episiotomy group ranked first, followed by the non-episiotomy group, and finally the routine episiotomy group. The network meta-analysis revealed non-significant effects between the non-episiotomy versus the routine episiotomy groups (RR = 0.57, 95% CI [0.24; 2.02]), and the restrictive episiotomy groups versus the routine episiotomy groups (RR = 0.50, 95% CI [0.14; 1.84]). The pooled studies were heterogenous (I² = 56.4%, *P* = 0.04) (Figs. 4E and 5E).

#### 4th degree perineal tear

##### Pairwise meta-analysis result

In two studies [39, 40] comparing the non-episiotomy and routine episiotomy groups, a non-significant risk reduction was found (RR = 0.88, 95% CI [0.19; 3.97]). Additionally, one study [31] comparing between the restrictive and routine episiotomy groups indicated a non-significant risk reduction (RR = 0.50, 95% CI [0.13; 2.00]) (Figs. 2F and 3F).

##### Network meta-analysis result and ranking

The restrictive episiotomy group ranked first, followed by the non-episiotomy group, and finally the routine episiotomy group. The network meta-analysis revealed non-significant effects between the non-episiotomy versus routine episiotomy groups (RR = 0.88, 95% CI [0.19; 3.97]), and the restrictive versus routine episiotomy groups (RR = 0.50, 95% CI [0.13; 2.00]). The pooled studies were homogeneous (I² = 12.4%, *P* = 0.29) (Figs. 4F and 5F).

### Secondary outcomes

We summarized the results of our secondary outcomes in Table 1. Also, we provide the detailed results of our secondary outcomes in the supplementary file from page 11 to page 21.

**Table 1:** Summary of our analysis

Outcome		Comparison	MA model	Number of studies	Estimate	95% CI	*P* value	Heterogeneity *P* value
Maternal outcomes	Post-partum blood loss > 500 ml	Restricted vs. no episiotomy	Direct	3	0.77	[0.47; 1.26]	-	0.46
			NMA	-	0.89	[0.57; 1.39]	0.61	
		Routine vs. no episiotomy	Direct	1	1.76	[0.89; 3.46]	-	
			NMA	-	1.34	[0.79; 2.27]	0.28	
		Restricted vs. routine episiotomy	Direct	1	0.88	[0.44; 1.76]	-	
			NMA	-	0.67	[0.39; 1.13]	0.13	
	Wound hematoma	No vs. restricted episiotomy	Direct	2	1.34	[0.25; 7.07]	-	0.48
			NMA	-	0.46	[0.21; 0.99]	0.05	
		No vs. routine episiotomy	Direct	1	0.33	[0.15; 0.71]	-	
			NMA	-	0.41	[0.20; 0.83]	0.01	
		Routine vs. restricted episiotomy	Direct	2	1.06	[0.72; 1.55]	-	
			NMA	-	1.12	[0.77; 1.63]	0.55	
	Wound infection	Restricted vs. no episiotomy	Direct	2	2.07	[0.79; 5.43]	-	0.62
			NMA	-	2.72	[1.13; 6.54]	0.03	
		Routine vs. no episiotomy	Direct	1	10.7	[1.51; 76.07]	-	
			NMA	-	3.44	[1.24; 9.58]	0.01	
		Restricted vs. routine episiotomy	Direct	3	0.92	[0.45; 1.88]	-	
			NMA	-	0.79	[0.40; 1.56]	0.02	
	Urinary incontinence	No vs. restricted episiotomy	Direct	2	0.64	[0.33; 1.25]	-	0.51
			NMA	-	0.64	[0.33; 1.25]	0.19	
		No vs. routine episiotomy	Direct	0	-	-	-	
			NMA	-	1.26	[0.21; 7.65]	0.79	
		Routine vs. restricted episiotomy	Direct	1	0.51	[0.10; 2.72]	-	
			NMA	-	0.51	[0.10; 2.72]	0.34	
	Anal incontinence	No vs. restricted episiotomy	Direct	1	0.31	[0.01; 7.65]	-	0.45
			NMA	-	0.12	[0.02; 1.00]	0.05	
		No vs. routine episiotomy	Direct	1	0.06	[0.01; 0.44]	-	
			NMA	-	0.09	[0.01; 0.50]	0.01	
		Routine vs. restricted episiotomy	Direct	1	1.02	[0.15; 7.10]	-	
			NMA	-	1.44	[0.26; 8.09]	0.68	
Neonatal outcomes	1st minute Apgar score < 7	No vs. restricted episiotomy	Direct	2	0.84	[0.49; 1.44]	-	0.79
			NMA	-	0.8	[0.48; 1.35]	0.41	
		No vs. routine episiotomy	Direct	1	0.36	[0.04; 3.37]	-	
			NMA	-	0.8	[0.44; 1.45]	0.46	
		Routine vs. restricted episiotomy	Direct	5	0.99	[0.73; 1.33]	-	
			NMA	-	1	[0.75; 1.35]	0.99	
	5-minute Apgar score < 7	No vs. restricted episiotomy	Direct	2	0.34	[0.05; 2.26]	-	0.88
			NMA	-	0.35	[0.06; 1.87]	0.22	
		No vs. routine episiotomy	Direct	1	0.35	[0.01; 8.73]	-	
			NMA	-	0.34	[0.04; 2.75]	0.31	
		Routine vs. restricted episiotomy	Direct	1	1.02	[0.14; 7.39]	-	
			NMA	-	1.01	[0.18; 5.80]	0.99	

*MA* Meta-analysis, *NMA* Network meta-analysis, *CI* Confidence interval.

## Discussion

### Summary of the findings

In our pairwise meta-analysis, comparisons between restrictive episiotomy and non-episiotomy groups, as well as routine episiotomy and non-episiotomy groups, showed non-significant effects across various parameters, including the duration of the 2nd stage, postpartum blood loss > 500 ml, wound hematoma, wound infection, postpartum perineal pain, urinary incontinence, anal incontinence, 1st, 2nd, and 3rd-degree perineal tears, and 1st and 5th-minute Apgar scores < 7. In contrast, our network meta-analysis found that routine episiotomy significantly increased the risk of wound infection, postpartum perineal pain, and anal incontinence compared to the non-episiotomy group, but significantly decreased the risk of 1st-degree perineal tears. Additionally, comparisons between restrictive and routine episiotomy groups showed non-significant effects across most parameters, except for a significant increase in the risk of 1st-degree perineal tears in the restrictive episiotomy group.

### Explanation of our finding

Our network meta-analysis reveals that routine episiotomy significantly increases the risk of wound infection, hematoma, postpartum perineal pain, and anal incontinence compared to the non-episiotomy group. This heightened risk is primarily due to the unnecessary trauma caused by episiotomy, which leads to greater pain and complications such as wound hematomas [44–46]. The disruption of the perineum’s natural tissue integrity, combined with the increased susceptibility to infection from the incision, further contributes to discomfort and delayed healing [47–49] Additionally, the impact of episiotomy on pelvic floor muscles may worsen anal incontinence [45]. These findings highlight the importance of reconsidering routine episiotomy practices to minimize unnecessary harm and optimize maternal health outcomes.

Perineal body and fibers of external anal sphincter are involved in the incisive plane of midline episiotomy in 14.3% and 16.6% of females, respectively. This specific anatomy may contribute to the higher incidence of postpartum perineal pain and anal incontinence in the episiotomy group. Additionally, the incisive plane of mediolateral episiotomy endangers superficial and deep perineal nerve branches, along with posterior labial nerves, further contributing to postpartum perineal pain [50]. Furthermore, hematomas may arise due to rupture pf subcutaneous blood vessels, taking into consideration the increased vascularity and perineal connective tissue softening during pregnancy [51, 52].

Moreover, our analysis showed no significant differences in most outcomes between restrictive and routine episiotomy groups. However, the restrictive approach was associated with a higher risk of 1st-degree perineal tears. This suggests that routine episiotomy, by providing a controlled incision, may help prevent spontaneous superficial lacerations. In contrast, the restrictive strategy, while reducing unnecessary incisions, may leave the perineum more susceptible to minor tears. Clinically, this highlights the need to balance minimizing episiotomy-related trauma with the potential risk of increased spontaneous perineal tears.

Current guidelines advocate for a restrictive approach, reserving episiotomy for necessary situations such as operative vaginal delivery, shoulder dystocia, or when managing a non-reassuring neonatal heart tracing [8]. Despite this, a systematic review on the efficacy of episiotomy for preventing and managing shoulder dystocia found no supporting evidence [53].

The American College of Obstetricians and Gynecologists (ACOG) acknowledges the lack of sufficient evidence-based criteria for episiotomy indications. The ACOG supports that clinical considerations should guide the decision-making process to perform an episiotomy and asserts that a restrictive approach is advisable [11]. Additionally, the WHO recommends against routine episiotomy during spontaneous vaginal delivery during spontaneous vaginal delivery [54]. Our findings go in alignment with WHO recommendations regarding the need to restrict routine episiotomy usage.

However, a global misinterpretation of evidence against episiotomies has led some physicians to avoid the procedure entirely, aiming to encourage the “naturalization” of delivery. This perspective has even labeled episiotomy as an “obstetrical violation” in certain institutions. Episiotomy, when performed based on indications and proper techniques, can be beneficial, preventing serious lacerations and expediting delivery in fetuses believed to be hypoxic. It is crucial to emphasize that while episiotomy may not be beneficial in all cases, failure to perform it when indicated can be equally detrimental [6].

Furthermore, key outcomes such as spontaneous laceration, the need for perineal suturing, suturing difficulty, dyspareunia, prolapse, maternal satisfaction, and wound dehiscence could not be fully assessed in our analysis due to insufficiently reported data in the included studies. These outcomes are essential to understanding the broader impact of perineal trauma and its management. Perineal injuries affect up to 90% of women during vaginal delivery and are associated with both short- and long-term complications, including persistent pain, dyspareunia, pelvic floor disorders, and depression [55]. Ensuring accurate assessment and appropriate repair of perineal trauma is crucial to reducing these complications [56]. By improving the reporting and analysis of these outcomes in future studies, we can enhance clinical decision-making and optimize patient care.

### Agreements and disagreements with previous studies

Our network analysis includes 16 studies, incorporating a substantial pooled sample size of 10,738 patients. In contrast, a meta-analysis by Pereira et al. [15] involved only 2 studies, compromising 546 patients, comparing restrictive episiotomy versus no episiotomy. Another Cochrane meta-analysis in 2017 [57] included 12 studies, compromising 6,177 patients, focusing on the comparison between restrictive and routine episiotomy.

Pereira et al. [15] and the Cochrane study [57] both observed no significant difference in perineal pain and postpartum blood loss between restrictive episiotomy and either no episiotomy or routine episiotomy, aligning with our results.

Concerning the duration of the 2nd stage, Pereira et al. [15] found no significant difference between restrictive episiotomy and no episiotomy. Similarly, the Cochrane study [57] reported no significant difference in 5-minute Apgar scores < 7 and urinary incontinence between restrictive and routine episiotomy. Both findings align with our results.

In terms of wound hematoma and wound infection, Rockner et al. [32] documented a noteworthy increase associated with routine episiotomy compared to no episiotomy. This finding is consistent with our results for wound hematoma but contradicts our results for wound infection. Additionally, studies by Ali et al. [28] and the Argentine Episiotomy Trial Collaborative Group [42] found no significant difference in wound hematoma and infection between routine episiotomy and restrictive episiotomy. Furthermore, Sagi-Dain 2017 [34] and Sagi-Dain 2021 [33], reported no significant difference in these parameters when comparing restrictive episiotomy to no episiotomy, consistent with our findings.

In terms of anal incontinence, Moini et al. [39] reported a significant increase with routine episiotomy compared to no episiotomy, consistent with our findings. Additionally, Murphy et al. [35], found no significant difference between routine episiotomy and restrictive episiotomy. Furthermore, Sagi-Dain [34] and Sagi-Dain [33], comparing restrictive episiotomy to no episiotomy, also found no significant difference, aligning with our results.This highlights the ongoing debate regarding the necessity of routine episiotomy and suggests that a more restrictive approach may be preferable to minimize complications.

Aquino et al. conducted a study to examine the incidence of obstetric anal sphincter injuries (OASIS) and identify potential risk factors [58]. They reported an OASIS incidence of 1.1%. Their analysis identified several significant risk factors for severe perineal tears, including nulliparity, previous cesarean section, use of assisted reproductive technology, weight gain during pregnancy, induced labor, oxytocin augmentation, epidural analgesia, gestation beyond 40 weeks, maternal position at delivery, labor duration, vacuum-assisted delivery, and newborn weight and head circumference [58]. These findings highlight the multifactorial nature of perineal trauma and underscore the importance of individualized risk assessment during labor and delivery.

Regarding first-degree perineal tear, Moini et al. [39] and Rockner et al. [32] found a significant decrease favoring routine episiotomy, similar to our results. Sajid et al. [40], however, found a non-significant difference, contrasting with our results. For second and third-degree perineal tear, Moini et al. [39] reported a significant increase with routine episiotomy, contrasting our findings, while Rockner et al. [32] and Sajid et al. [40] found non-significant differences, aligning with our results. For fourth-degree perineal tear, both Moini et al. [39] and Sajid et al. [40] reported non-significant differences, in line with our findings.

Moreover, in studies by Eltorky et al. [37], Sulaiman et al. [30], and House et al. [29] comparing routine episiotomy to restrictive episiotomy, Sulaiman et al. [30] and House et al. [29] found a significant decrease favoring routine episiotomy, aligning with our findings. Eltorky et al. [27], however, found a non-significant difference, contrasting with our results. For second-degree perineal tear, Eltorky et al. [37] and House et al. [29] reported a significant decrease favoring routine episiotomy, in contrast to our findings, while Sulaiman et al. [30] found a significant increase with routine episiotomy, also contrasting with our findings. In third-degree perineal tear, studies by Rodriguez et al. [30, 31], Sulaiman et al. [30], and House et al. [29] found a non-significant difference, aligning with our findings. For fourth-degree tear, Rodriguez et al. [31] found a non-significant difference, aligning with our findings. Sagi-Dain et al. 2017 [34], in their study comparing restrictive episiotomy to non-episiotomy, found a non-significant difference in first, second, third, and fourth-degree perineal tear which align with our finding.

### Strength points and limitations

Currently, this study represents the most comprehensive network meta-analysis comparing non-episiotomy, routine episiotomy, and restrictive episiotomy in primiparous women in relation to maternal and infant outcomes. We considered RCTs only to provide strong evidence. Encompassing 16 clinical trials with a total of 10,738 patients, we systematically evaluated 11 maternal outcomes and 2 neonatal outcomes, providing a robust overview of the current evidence.

Despite these strengths, our study has several limitations, which warrant acknowledgment. Firstly, the analysis was confined to studies conducted in English, potentially introducing a publication bias, and limiting the generalizability of our findings to a more diverse linguistic context. Secondly, we identified heterogeneity among the included trials, which may impact the consistency and general applicability of our findings. Furthermore, certain outcomes, including spontaneous laceration, the need for perineal suturing, suturing difficulty, pain scores, time to return to normal activities, dyspareunia, prolapse, maternal satisfaction, and wound dehiscence, could not be assessed in our analysis due to insufficiently reported data in the included studies. Also, we could not perform a subgroup analysis based on episiotomy type due to inconsistent reporting across studies. Even when the type was reported, the outcomes were not consistently included, potentially introducing confounding bias since complications differ between midline and mediolateral episiotomies. However, within each study, this variation was controlled. For instance, studies using midline episiotomies in the routine group also used them in the restrictive group, and the same was true for mediolateral episiotomies. This consistency helped significantly reduce bias. This limitation underscores the incompleteness of the available evidence and emphasizes the importance of more comprehensive reporting in future studies.

### Implications of our findings in practice

In our network meta-analysis, the routine episiotomy group exhibited significantly higher impacts on wound hematoma, wound infection, post-partum perineal pain, and anal incontinence, indicating potential drawbacks associated with this approach. Conversely, the restrictive episiotomy group demonstrated non-significantly higher effects than the non-episiotomy group, except for wound infection. These findings underscore the need for a reevaluation of the current practices surrounding episiotomies, encouraging a more restrictive and individualized approach based on the specific needs and conditions of each patient. Healthcare providers should consider these results when making decisions regarding episiotomy procedures, aiming to optimize maternal and neonatal outcomes while minimizing unnecessary interventions and associated complications.

### Recommendations

Future research in the field of episiotomy should prioritize several aspects to enhance our understanding and improve obstetric care practices. First and foremost, there is a need for well-designed, large-scale studies with rigorous methodologies to provide more robust evidence on the impact of episiotomy on maternal and neonatal outcomes. These studies should encompass diverse populations to ensure the generalizability of findings. Including outcomes such as spontaneous laceration, the need for perineal suturing, suturing difficulty, pain scores, time to return to normal activities, dyspareunia, prolapse, maternal satisfaction, and wound dehiscence in future research would provide a more comprehensive understanding of the procedure’s implications. Moreover, exploring the long-term effects of episiotomy on maternal and neonatal health could guide clinical decision-making. Also, future research should address the limitations of inconsistent reporting for episiotomy types. Standardizing the reporting of episiotomy types would enable robust subgroup analyses and clarify the distinct complications associated with midline versus mediolateral episiotomies. Lastly, assessing the impact of cultural and contextual factors on episiotomy rates and outcomes may help tailor recommendations for different settings. Overall, future research endeavors should strive for methodological rigor, inclusivity, and a holistic examination of the various facets of episiotomy to inform evidence-based obstetric care. Future research should aim to include studies in multiple languages to enhance the external validity of the results.

## Conclusion

Non-episiotomy approaches consistently perform best, reducing the risk of complications like wound hematoma and infection. Routine episiotomy, on the other hand, shows higher risks, especially in wound infection. While restrictive episiotomy fares better than routine, it still lags behind non-episiotomy methods. In essence, non-episiotomy strategies are preferred, followed by restrictive episiotomy, with routine episiotomy being the least favorable. Nonetheless, individual patient factors should guide treatment decisions, and more research is needed to validate these findings and refine clinical practices.

## Supplementary Information

Supplementary Material 1.

## Data Availability

No datasets were generated or analysed during the current study.
