# Supplementary material for: Network meta-analysis of episiotomy approaches: comparing routine, restrictive, and non-episiotomy strategies and their effects on maternal and neonatal outcomes in primiparous patients
Source: Reprod Health. 2025 Sep 16;22:161. doi: 10.1186/s12978-025-02132-9 (PMC12442280; doi:10.1186/s12978-025-02132-9)
Supplement: Supplementary file 1 — Supplementary Material 1. [file 12978_2025_2132_MOESM1_ESM.docx]

**Supplementary materials**

**Supplementary Table 1** Search terms and results in different databases.

| **Database** | **Search strategy** | **Filter** | **Results** | **Search date** |
| --- | --- | --- | --- | --- |
| PubMed | (Episiotomy OR episiotomies OR Perineotomy OR perineotomies OR "Episiotomy"[Mesh] OR non-episiotomy) | All Fields | 4131 | March 25, 2024 |
| Web of Science | (Episiotomy OR episiotomies OR Perineotomy OR perineotomies OR “non-episiotomy”) | Title and Abstract | 3464 |  |
| Scopus |  |  | 6373 |  |
| Cochrane | 1- Episiotomy OR episiotomies OR Perineotomy OR perineotomies OR medio-lateral episiotomy OR Midline episiotomy OR Restrictive episiotomy OR Restrictive episiotomies OR routine episiotomy OR routine episiotomies OR Restricted episiotomy OR selective episiotomies OR Lateral episiotomy OR Lateral episiotomies OR median episiotomy OR J-shaped episiotomy OR "Episiotomy"[Mesh] 2- zero episiotomy OR non-episiotomy OR no episiotomy OR non episiotomy OR null episiotomy 3- #1 OR #2 | NA | 1639 |  |

NA, not applicable.

**Supplementary Table 2** Summary of the included studies.

| study ID | Type of episiotomy | | Country, site involved | Sample size | Inclusion criteria | Exclusion criteria | study duration | conclusion |
| --- | --- | --- | --- | --- | --- | --- | --- | --- |
|  | **Intervention** | **control** |  |  |  |  |  |  |
| Amorim et al. 2017 | Restricted episiotomy | No episiotomy | Recife, Northeastern Brazil. | 263 | with clinically stable women in active labor with a live, full-term fetus (37 to 41 weeks of pregnancy) in cephalic presentation (vertex position), and with dilatation of 6 to 8 cm. | Exclusion criteria consisted of pregnancy bleeding disorders (premature detachment of the placenta); indication for a cesarean section; cephalopelvic disproportion, non-reassuring fetal heart rate, dystocia; women incapable of giving their consent, and women with no responsible accompanying person. In addition, women who were submitted to a cesarean section after enrolment in the study were excluded (post-randomization exclusion). | From July to September 2014. | An overall episiotomy rate of less than 2% was found in the two groups evaluated in the present study, showing that it is possible to implement a non-episiotomy protocol for a group of women with full-term pregnancies and fetuses in the cephalic position without any statistically significant differences in relation to the selective practice of the procedure. |
| Moini et al. 2009 | Routine episiotomy | No episiotomy | The Arash Hospital, in Tehran, Iran | 283 | Primiparous women with a full-term singleton pregnancy and no underlying problems. | Were non-cephalic and cephalic presentations except occiputo-anterior and fetal macrosomia. | From April 2007 until March 2008. | The present study demonstrated that routine episiotomy is associated with an increased risk of third- and fourth-degree tears and subsequent complications especially pain, dyspareunia, and incontinence. |
| Rockner et al. 1988 | Mediolateral episiotomy | No episiotomy | Department of Obstetrics, Huddinge University Hospital, | 205 | 218 years of age, full-term (237 weeks of gestation), singleton delivery and Swedish nationality. No immigrants were included because of the language limitations. Only women with ST (without Ep) of at least 2cm in length of the perineum were included. | There were two refusals, due to tiredness after delivery and personal problems. | One year. | Furthermore, delayed healing was recorded for the episiotomy group and more analgesia was used post-partum by women with episiotomies. |
| Sagi-Dain et al. 2017 | Restricted episiotomy  (lateral and Mediolateral) | No episiotomy | Bnai-Zion Medical Center, Haifa, Israel. | 311 | We included women experiencing their first vaginal delivery (including trial of labor after cesarean section), with a singleton pregnancy of > 34 gestational weeks, vertex presentation, with no absolute contraindications for vaginal delivery. | Patients asked to be excluded. | From 31 May 2015 to  30 May 2016. | No difference in the rates of advanced perineal tears was found between groups; however, the main limitation of our study was unexpectedly high rates of episiotomy in the non-episiotomy group. Thus, the main conclusion is that investigator monitoring, and education should be continuously practiced throughout the trial duration, stressing the importance of adherence to the protocol. |
| Sagi-Dain et al. 2021 | Restricted episiotomy (Mediolateral) | No episiotomy | Tertiary medical center. | 525 | Inclusion criteria were women expecting their first vaginal delivery, with a singleton pregnancy, gestational age ≥ 34 weeks, vertex presentation, and lack of absolute contraindications to vaginal delivery. | NA | From May 2015  To May 2018. | In women with non-operative vaginal delivery, suturing of spontaneous perineal tears was easier and shorter compared to episiotomy repair. This might be related to the unpredictable nature of perineal tears, which might be shorter and shallower compared to the standard episiotomy incision. |
| Sagi-Dain et al. 2020 | Restricted episiotomy | No episiotomy | Bnai-Zion Medical Center— one of the three major hospitals in Haifa | 692 | The study group in which episiotomy was allowed only in cases of fetal distress) or “standard care. | were excluded before the allocation | Between May 31, 2015  and  May 6, 2018. | Since decreased use of episiotomy was not associated with higher rates of severe tears or any other adverse outcomes, we believe this procedure can be avoided in spontaneous as well as vacuum-assisted deliveries. |
| Sajid et al. 2019 | Mediolateral episiotomy | No episiotomy | Gynae unit 5, KEMU, Lady Aitchison hospital, Lahore | 180 | 1_primigravida females of age 18_40,2_ presenting at term 3_ singleton cephalic pregnancy of fetal weight ranges from 2.5 kg to 3.5 kg spontaneous vaginal delivery. | Females with multiple pregnancies, Macrosomia fetal weight more than 4 kg, Mal-presentation, malposition, Gestational, chronic systemic problems. Diabetes, pregnancy-induced hypertension, and pre-eclampsia. Females with a history of extended perineal injuries or surgery were also Excluded. | one year 1_7_2015 to 30_6_2016 | According to our study, the incidence of perineal tear was 29.no significant association was found between groups and the degree of perineal tear. |
| Sleep et al. 1984 | Routine episiotomy (Mediolateral) | Restricted episiotomy  (Mediolateral) | Maternity Unit, Royal Berkshire Hospital | 1077 | They had alive singleton fetuses of at least 37 completed weeks' gestational age presenting cephalically, and spontaneous vaginal delivery was expected towards the end of the second stage of labor. | restrict episiotomy to fetal indications (fetal bradycardia, tachycardia, or meconium-stained liquor). | A five-month study period  in 1982. | Women allocated to the restrictive policy were more likely to have resumed sexual intercourse within a month after delivery. These findings provide little support either for liberal use of episiotomy or for claims that reduced use of the operation decreases postpartum morbidity. |
| Sulaiman et al. 2013 | Routine episiotomy (Mediolateral) | Restricted episiotomy (Mediolateral) | Tertiary hospital of the Universiti Kebangsaan Malaysia Medical Center, Kuala Lumpur, Malaysia | 171 | Live singleton pregnancy with a cephalic presentation, gestation beyond 37 weeks, primigravida, women with no history of severe perineal injuries, and no life-threatening medical or psychiatric conditions. | Women with, multiple pregnancy, fetal mal-presentation, and delivery conducted by house officers and junior midwives were excluded. | Between May and October 2009. | Routine Mediolateral episiotomy in primigravida is associated with a higher prevalence of obstetrical anal sphincter injuries. As anal sphincter injuries are known to have morbidities, selective Mediolateral episiotomy in primigravida is therefore recommended in the implementation of the new delivery practice, and in an attempt to reduce our high episiotomy rate. |
| House et al. 2015 | Routine episiotomy (Mediolateral) | Restricted episiotomy  (Mediolateral) | Charing Cross Hospital Medical School, West London Hospital, London | 165 patients | In the patients in whom the use of episiotomy was restricted (episiotomies 32 percent in primigravida, 2 percent in multigravida), 32 percent of primigravida and 54 percent of multigravida had intact perineum or a first-degree tear. | Exclusion criteria were lack of consent, labor at less than 37 weeks pregnant, presentation other than vertex, cesarean section, and the unavailability of an accouter willing to abide by the research protocol. | 12-month period. | The study failed to reveal any differences in long-term follow-up between a liberal and restrictive use of episiotomy. A restrictive policy resulted in a significant increase in the incidence of patients with intact perineum or only a first-degree tear (32 as against 4 percent in primipara and 54 as against 26 percent in multipara. |
| Murphy et al. 2008 | Routine episiotomy | Restricted episiotomy | The maternity unit in Scotland  and  Southwest England | 317 women | The sample comprised nulliparous women expected to deliver at ‡37 weeks of gestation with live singleton cephalic pregnancies and no contraindication to vaginal birth. | We excluded women who were less than 16 years or with limited ability to speak or understand English. | The maternity unit in Scotland from October 2004 to September 2006.  The unit in Southwest England from June 2005 to August 2006. | This pilot study does not provide conclusive evidence that a policy of routine episiotomy is better or worse than a restrictive policy. A definitive RCT is feasible but will require a large sample size to inform clinical practice. |
| Rodriguez et al. 2008 | Routine episiotomy (Midline) | Restricted episiotomy  (Midline) | San Vicente de Paul teaching hospital, a high complexity care level institution in Medellín Antioquia | 446 | Nulliparous women with pregnancies more than 28 weeks of gestation who had vaginal deliveries. | Women with multiple pregnancies were excluded, as were patients with breech presentations and those who did not sign the informed consent or refused to participate in the study. | Between February 2002 and November 2004. | The policy of performing selective Midline episiotomy in nulliparous patients results in a reduction in the risk of third-degree perineal lacerations. |
| Sangkomkamhang et al. 2019 | Routine episiotomy (Median or Mediolateral) | Restricted episiotomy  (Median or Mediolateral) | Thailand Srinagarind Hospital  (Khon Kaen University) and Khon Kaen Hospital, and two general hospitals (Kalasin Hospital and Lamphun Hospital). | 3006 | 3006 singleton pregnant women 18 years or older, ≥37 weeks of gestation, cephalic presentation, and planned vaginal delivery. | Pregnant women with multiple gestations were excluded, as were women with breech presentation or bleeding disorders and those admitted for planned cesarean section. | Between September 2015 and December 2017. | Restrictive episiotomy results in more intact perineum in multiparous women. Risks of maternal and neonatal outcomes were comparable between the two practices. These results strengthen the certainty of the existing Cochrane review findings in supporting restrictive episiotomy. |
| Ali et al. 2004 | Routine episiotomy (Mediolateral) | Restricted episiotomy  (Mediolateral | Jinnah hospital, Lahore, Pakistan | 200 | Primigravida in labor at term with a singleton fetus in cephalic presentation, patients with growth fetal malformations. | Don't have the criteria. | NA | Study that there is no evidence that routine use of episiotomy has any beneficial effect. On contrary, there is clear evidence that it may cause more harm in the form of damage to the perineum with a greater need for surgical repair. |
| Argentine Episiotomy Trial Collaborative Group | Routine episiotomy (Mediolateral) | Restricted episiotomy  (Mediolateral) | In 8 city public maternity hospitals in Argentina, | 2606 | Women were eligible if they were in uncomplicated labor at 37 to 42 weeks, nulliparous or primiparous gestation, with a single fetus in cephalic presentation, and had no history of cesarean delivery or severe perineal tears. | Who didn't have the criteria for entry were excluded. | From August 1990 to July 1992. | Anterior perineal trauma was more common in the selective group but posterior perineal surgical repair, perineal pain, healing complications, and dehiscence were all less frequent in the selective group. Routine episiotomy should be abandoned and episiotomy rates above 30% cannot be justified. |
| Eltorky et al. 1994 | Routine episiotomy (Mediolateral) | Restricted episiotomy  (Mediolateral) | Maternity Unit at the Riyadh Armed Forces Hospital, Saudi Arabia. | 200 | Live singleton fetus of at least 37 weeks gestational age, presenting cephalically, not suffering from any important medical or psychiatric condition and spontaneous vaginal delivery was expected towards the end of the second stage of labor. | Who didn't have the criteria for entry were excluded. | Between June and September 1993. | No difference was found between the two groups in either para-urethral lacerations or lateral vaginal wall lacerations. We favor the selective use of episiotomy in primigravida patients. |
|  |  |  |  |  |  |  |  |  |
|  |  |  |  |  |  |  |  |  |

**Supplementary Table 3** Baseline characteristics of patients in the included studies.

| Study ID | Intervention | | Control | | Mother’s age, years  Mean (SD) | | Gestational age at delivery, weeks Mean (SD) | | Birth weight  Mean (SD) | | BMI, kg/m2  Mean (SD) | |
| --- | --- | --- | --- | --- | --- | --- | --- | --- | --- | --- | --- | --- |
|  | Type | No. of patients | Type | No. of patients | Intervention | Control | Intervention | Control | Intervention | Control | Intervention | Control |
| Amorim et al. 2017 | Restricted episiotomy | 122 | No episiotomy | 115 | 23.5 (5.6) | 23.9 (6.3) | 38.67 (0.75) | 39.17 (1.88) | 3259.8 (398.4) | 3283.7 (408.1) | NA | NA |
| House et al. 2015 | Routine episiotomy (Mediolateral) | 71 | Restricted episiotomy  (Mediolateral) | 94 | NA | NA | NA | NA | 3400 (429) | 3282 (399) | NA | NA |
| Moini et al. 2009 | Routine episiotomy | 146 | No episiotomy | 137 | 24.22 (4.41) | 23.25 (5) | NA | NA | 3158.22 (345.81) | 3238.91 (399.99) | 27.63 (1.87) | 28.45 (2.48) |
| Murphy et al. 2008 | Routine episiotomy | 99 | Restricted episiotomy | 101 | NA | NA | 40.43 (9) | 40.57  (10) | 3589 (524) | 3550 (501) | NA | NA |
| Rockner et al. 1988 | Mediolateral episiotomy | 157 | No episiotomy | 48 | NA | NA | NA | NA | NA | NA | NA | NA |
| Rodriguez et al. 2008 | Routine episiotomy (Midline) | 223 | Restricted episiotomy  (Midline) | 222 | 19.7 (4) | 19.8 (4.1) | 38  (2.1) | 38 (2.1) | 2936 (470) | 289x9 (481) | NA | NA |
| Sagi-Dain et al. 2017 | Restricted episiotomy  (lateral and Mediolateral) | 155 | No episiotomy | 154 | 28.3 (4.6) | 28.7 (4.1) | 39.8 (1.2) | 39.5 (1.2) | 3285.8 (413.6) | 3228.4 (451.7) | 28.1  (4.8) | 27.5  (4.6) |
| Sagi-Dain et al. 2021 | Restricted episiotomy (Mediolateral) | 165 | No episiotomy | 270 | 28.4 (4.1) | 28 (4.3) | 39.7 (1.1) | 39.6 (1.2) | 3294.8 (386.5) | 3303.7 (436.9) | 27.8  (4.6) | 27.8  (4.4) |
| Sagi-Dain et al. 2020 | Restricted episiotomy | 339 | No episiotomy | 337 | 28.3 (4.6) | 28.4 (4) | 39.7 (1.2) | 39.5 (1.2) | 3269.5 (430.5) | 3297.4 (451.4) | 28  (4.7) | 27.7  (4.5) |
| Sajid et al. 2019 | Mediolateral episiotomy | 90 | No episiotomy | 90 | 28.47 (6.07) | 28.4 (6.62) | 39.58 (1.13) | 39.34 (1.15) | NA | NA | NA | NA |
| Sangkomkamhang et al. 2019 | Routine episiotomy (Median or Mediolateral) | 1504 | Restricted episiotomy  (Median or Mediolateral) | 1502 | 26.5 (5.9) | 26.7 (5.8) | 38.6 (1.2) | 38.5 (1.1) | 3081 (353.1) | 3081.8 (370.1) | 26.8  (4) | 26.8  (4.1) |
| Sleep et al. 1984 | Routine episiotomy (Mediolateral) | 502 | Restricted episiotomy  (Mediolateral) | 498 | 26.7 (5.3) | 26.6 (5.2) | 39.8 (1.2) | 39.8 (1.2) | 3367 (438) | 3393 (448) | NA | NA |
| Sulaiman et al. 2013 | Routine episiotomy (Mediolateral) | 82 | Routine episiotomy (Mediolateral) | 89 | 26.2 (4.12) | 26 (3.05) | NA | NA | NA | NA | 25.45 (18.57) | 24.94 (23.54) |
| Ali et al. 2004 | Routine episiotomy (Mediolateral) | 100 | Restricted episiotomy  (Mediolateral) | 100 | 24.86 (3.39) | 24.96 (3.33) | 38.87 (0.78) | 38.85 (0.79) | 2982.9 (313.9) | 3021 (323.3) | NA | NA |
| Argentine Episiotomy Trial Collaborative Group | Routine episiotomy (Mediolateral) | 1298 | Restricted episiotomy  (Mediolateral) | 1308 | NA | NA | NA | NA | 3244 (418.3) | 3244 (427.3) | NA | NA |
| Eltorky et al. 1994 | Routine episiotomy (Mediolateral) | 100 | Restricted episiotomy  (Mediolateral) | 100 | 21 (3.5) | 21.2 (3.9) | 39.86 (1.14) | 40 (1.29) | 3080 (399) | 3069 (438) | NA | NA |

SD, standard deviation; Na, not applicable.


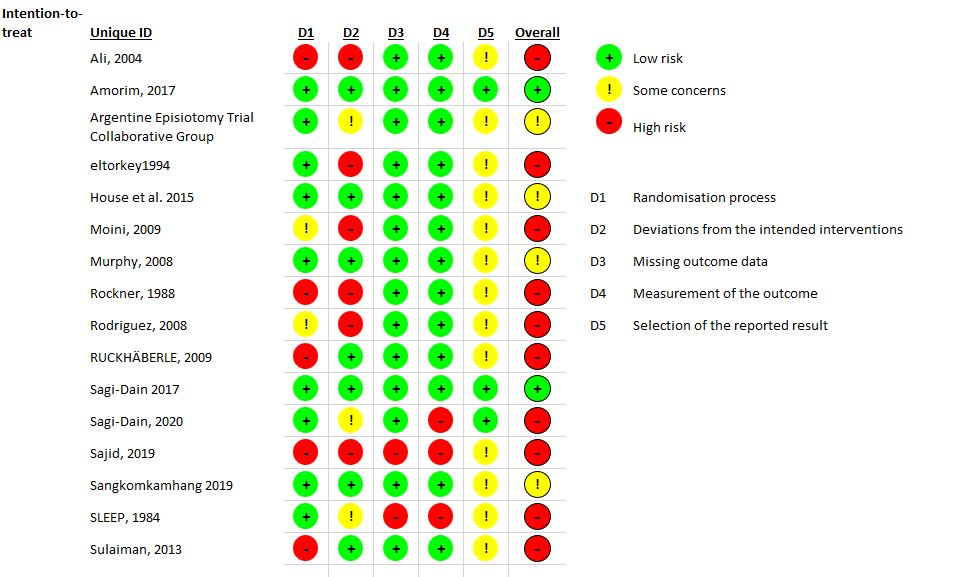


**Supplementary Figure S1A** Risk of bias summary: review authors' judgments about each risk of bias item for each included study.

**Supplementary Figure S1B** Risk of bias graph: review authors' judgments about each risk of bias item presented as percentages across all included studies.

# 3.4.1 post-partum blood loss > 500 ml

## 3.4.1.1 Pairwise meta-analysis result

In three studies (1–3) comparing the restrictive and non-episiotomy groups, a non-significant risk reduction was observed (RR = 0.77, 95% CI [0.47, 1.26]). Similarly, one study (4) comparing between the routine and non-episiotomy groups found a non-significant difference (RR = 1.76, 95% CI [0.89, 3.46]). Finally, in one study (5) comparing the restrictive and routine episiotomy groups, a non-significant risk reduction was found (RR = 0.88, 95% CI [0.44, 1.76]) (**Supplementary Figures S2** and **S16A**)


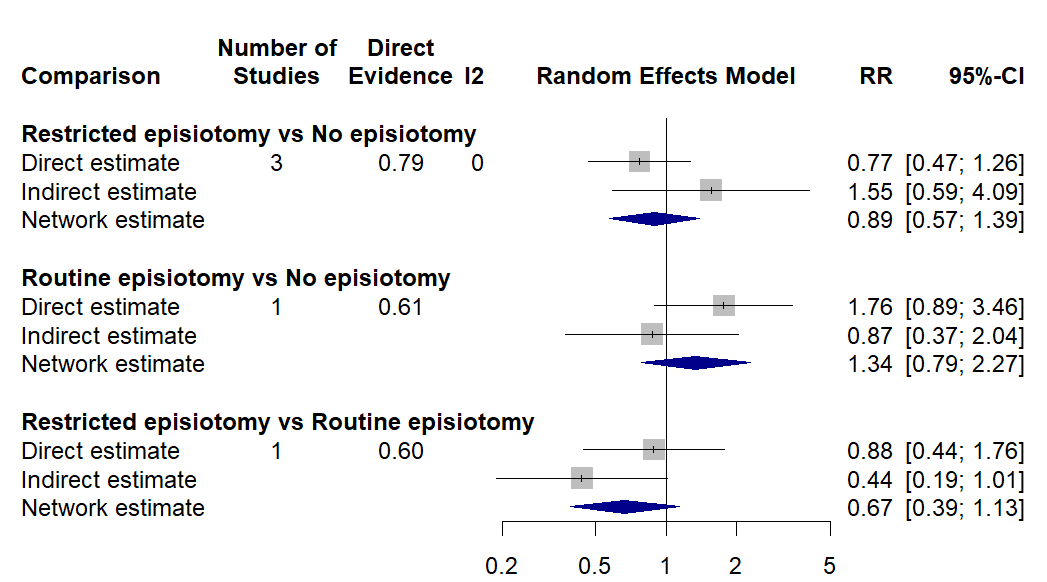


**Supplementary Figure S2** Pairwise meta-analysis for post-partum blood loss > 500 ml.

## 3.4.1.2 Network meta-analysis result and ranking

The restrictive episiotomy ranked first, followed by the non-episiotomy, and finally, routine episiotomy group. The network meta-analysis revealed non-significant effects between the routine episiotomy and non-episiotomy groups (RR = 1.34, 95% CI [0.79, 2.27]), and between the restrictive and non-episiotomy groups (RR = 0.89, 95% CI [0.57, 1.39]). The pooled studies were homogeneous (I² = 0%, *P* = 0.46) (**Supplementary Figure S3** and **S17A**)


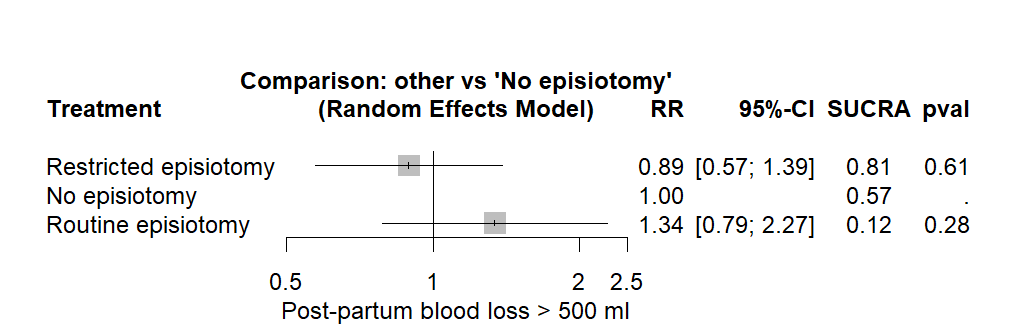


**Supplementary Figure S3** Forest plot of network meta-analysis for post-partum blood loss > 500 ml.

# **3.4.2 Wound hematoma**

## 3.4.2.1 Pairwise meta-analysis result

In two studies (2,3) comparing the non-episiotomy and restrictive episiotomy groups, a non-significant higher risk was observed (RR = 1.34, 95% CI [0.25; 7.07]). Similarly, two studies (6,7) comparing between the routine and restrictive episiotomy groups found a non-significant risk reduction (RR = 1.06, 95% CI [0.72; 1.55]). In contrast, in one study (4) comparing between the non-episiotomy and routine episiotomy groups, a significant risk reduction was found (RR = 0.33, 95% CI [0.15; 0.71]) (**Supplementary Figure S4** and **S16B**).


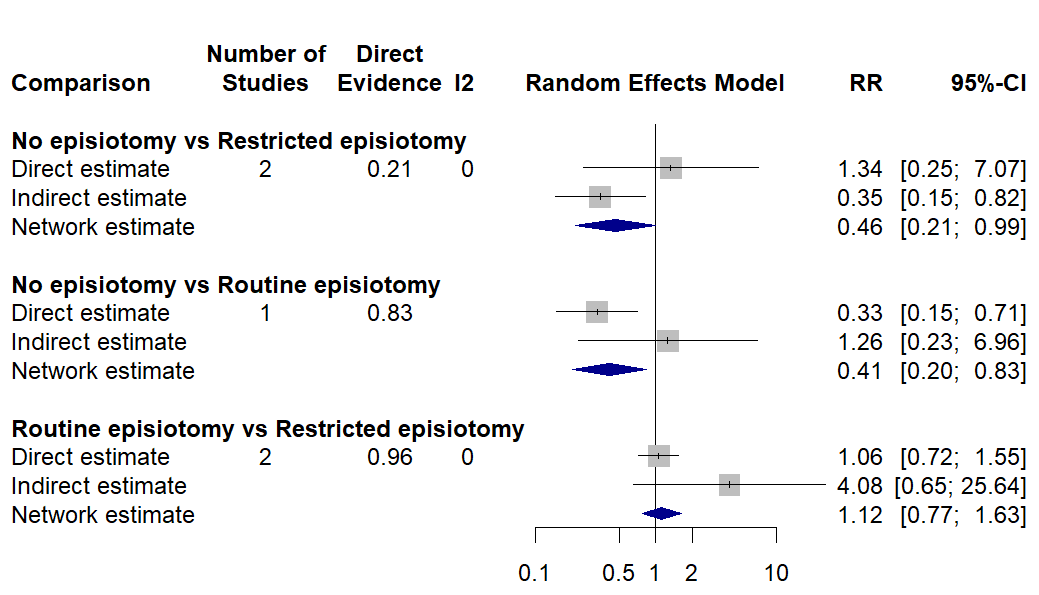


**Supplementary Figure S4** Pairwise meta-analysis for wound hematoma.

## 3.4.2.2 Network meta-analysis result and ranking

The non-episiotomy ranked first, followed by the restrictive, and finally, routine episiotomy group. Interestingly, only the non-episiotomy group demonstrated a significantly lower risk than the restrictive episiotomy group (RR = 0.46, 95% CI [0.21; 0.99], *P* = 0.05). In contrast, routine episiotomy showed a non-significantly higher risk than restrictive episiotomy (RR = 1.12, 95% CI [0.77; 1.63]). The pooled studies were homogeneous (I² = 0%, *P* = 0.48) (**Supplementary Figure S5** and **S17B**).


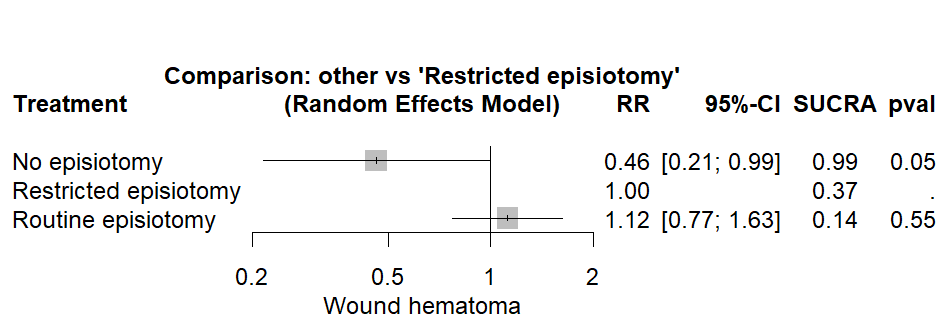


**Supplementary Figure S5** Forest plot of network meta-analysis for wound hematoma.

# 3.4.3 Wound infection

## 3.4.3.1 Pairwise meta-analysis result

In two studies (2,3) comparing the restrictive and non-episiotomy groups, a non-significant higher risk reduction was observed with the restrictive episiotomy (RR = 2.07, 95% CI [0.79, 5.43]). Conversely, in one study (4) comparing between the routine and the non-episiotomy group, a significant increase in risk was observed with the routine episiotomy group (RR = 10.70, 95% CI [1.51,76.07]). Additionally, three studies (6–8) comparing between the restrictive and routine episiotomy groups indicated a non-significant risk reduction (RR = 0.92, 95% CI [0.45, 1.88]) (**Supplementary Figures S6** and **S16C**).

**
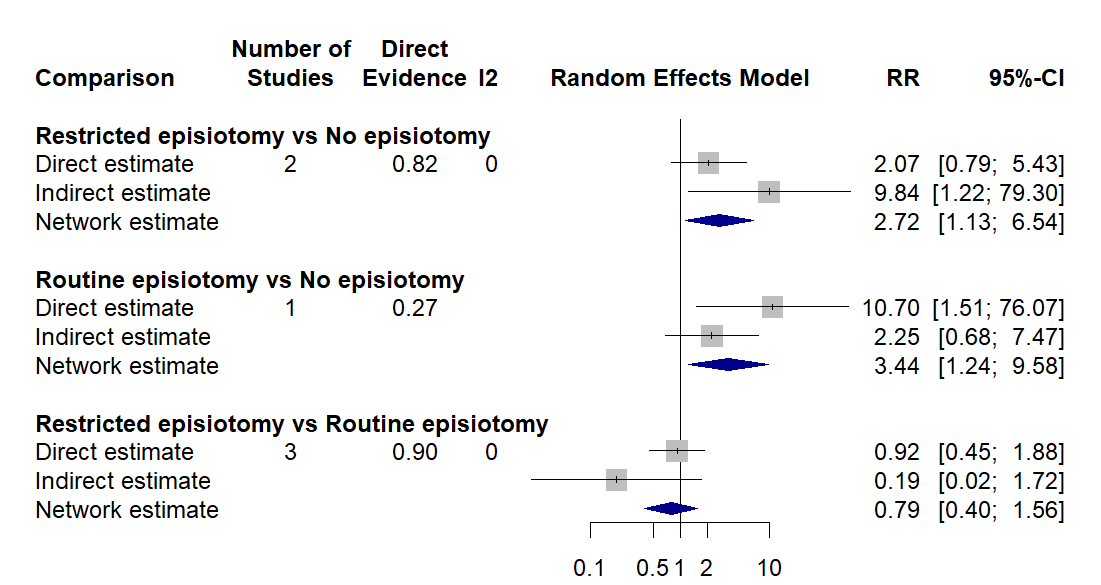
**

**Supplementary Figure S6** Pairwise meta-analysis for wound infection.

## **3.4.3.2 Network meta-analysis result and ranking** The non-episiotomy ranked first, followed by restrictive, and finally, routine episiotomy group. Notably, routine episiotomy group exhibited a significantly higher risk than non-episiotomy (RR = 3.44, 95% CI [1.24, 9.58], *P* = 0.02). Similarly, restrictive episiotomy exhibited a significantly higher risk than non-episiotomy (RR = 2.72, 95% CI [1.13; 6.54], *P* = 0.03). The pooled studies were homogeneous (I² = 0%, *P* = 0.62) (Supplementary Figure S7 and S17C).

**
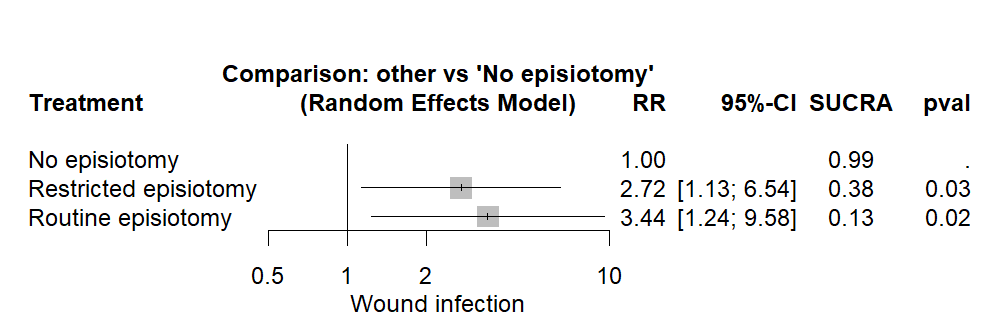
**

**Supplementary Figure S7** Forest plot of network meta-analysis for wound infection.

# 3.4.4 Urinary incontinence

## 3.4.4.1 Pairwise meta-analysis result

In two studies (2,3) comparing the non-episiotomy and restrictive episiotomy groups, a non-significant risk reduction was observed (RR= 0.64, 95% CI [0.33; 1.25]). Additionally, in one study (9) comparing between the routine and restrictive episiotomy groups, a non-significant risk reduction was found (RR = 0.51, 95% CI [0.10; 2.72]) (**Supplementary Figures S8** and **S16D**).


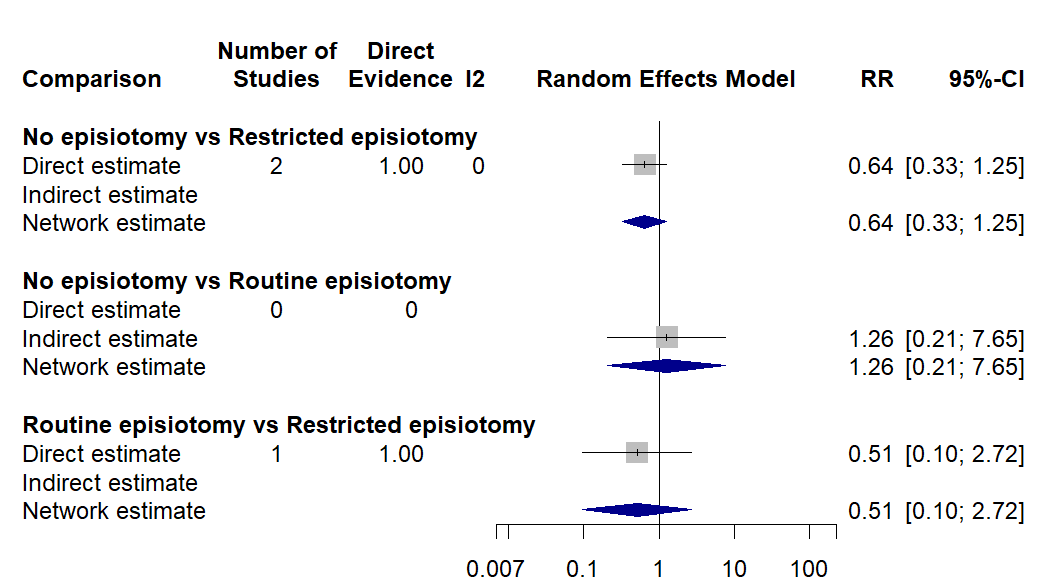


**Supplementary Figure S8** Pairwise meta-analysis for urinary incontinence.

## **3.4.4.2 Network meta-analysis result and ranking** The routine episiotomy ranked first, followed by the non-episiotomy, and finally, restrictive episiotomy. Both routine and non-episiotomies exhibited a non-significantly lower risk than restrictive episiotomy (RR = 0.51, 95% CI [0.10; 2.72]; RR = 0.64, 95% CI [0.33, 1.25]). The pooled studies were homogeneous (I² = 0%, *P* = 0.51) (Supplementary Figures S9 and S17D).


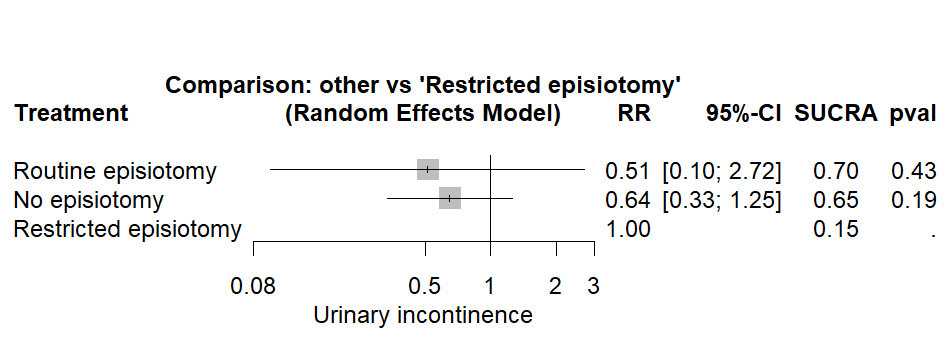


**Supplementary Figure S9** Forest plot of network meta-analysis for urinary incontinence.

# 3.4.5 Anal incontinence

## 3.4.5.1 Pairwise meta-analysis result

In one study (3) comparing non-episiotomy and restrictive episiotomy, a non-significant risk reduction was observed (RR = 0.31, 95% CI [0.01; 7.65]). Similarly, one study (9) comparing between routine and restrictive episiotomy indicated a non-significant difference (RR = 1.02, 95% CI [0.15; 7.10]). Conversely, in one study (10) comparing between non-episiotomy and routine episiotomy, a significant decrease in risk was observed with the non-episiotomy group (RR = 0.06, 95% CI [0.01; 0.44]) (**Supplementary** **Figures S10** and **S16E**).


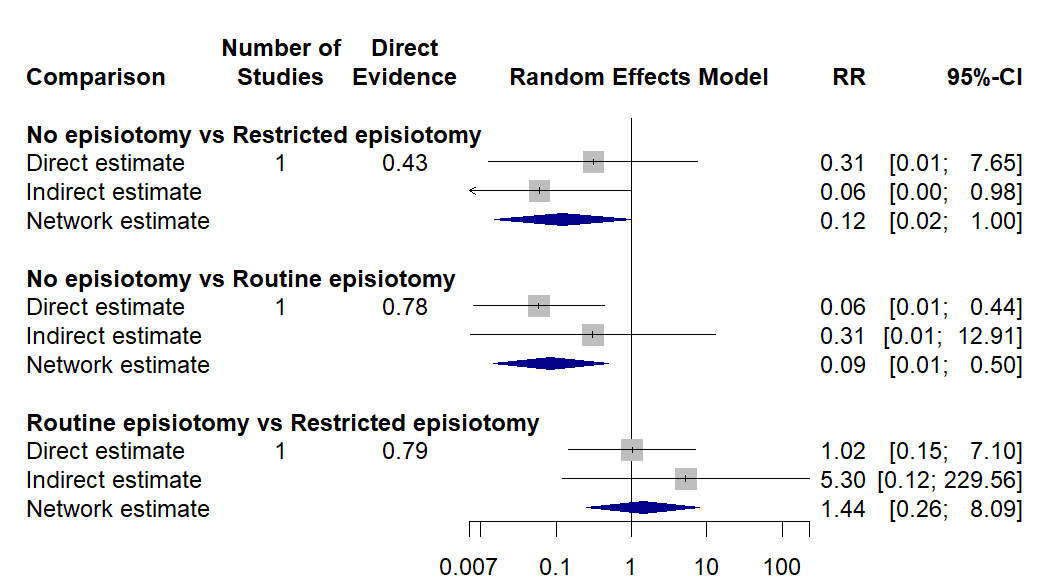


**Supplementary Figure S10** Pairwise meta-analysis for anal incontinence.

## 3.4.5.2 Network meta-analysis result and ranking

Non-episiotomy ranked first, followed by restrictive, and finally, routine episiotomy. Both non- episiotomy and routine episiotomy were comparable to restrictive episiotomy (RR = 0.12, 95% CI [0.02; 1.00]; RR = 1.44, 95% CI [0.26; 8.09], respectively). The pooled studies were homogeneous (I² = 0%, *P* = 0.45) (Supplementary **Figures S11** and **S17E**).


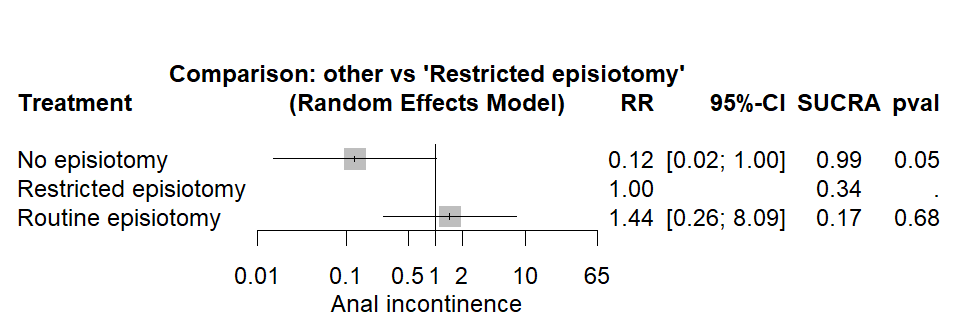


**Supplementary Figure S11** Forest plot of network meta-analysis for anal incontinence.

# 3.4.6 1st minute Apgar score <7

## 3.4.6.1 Pairwise meta-analysis result

In two studies (1,3) comparing 1st minute Apgar score <7 incidence between non-episiotomy and restrictive episiotomy, a non-significant risk reduction was observed (RR = 0.84, 95% CI [0.49; 1.44]). Similarly, in one study (10) comparing between non-episiotomy and routine episiotomy, a non-significant risk reduction was found (RR = 0.36, 95% CI [0.04; 3.37]). Additionally, five studies (5,7,9,11,12) between routine and restrictive episiotomies indicated a non-significant risk reduction (RR = 0.99, 95% CI [0.73; 1.33]) (**Supplementary Figures S12** and **S16F**).

**
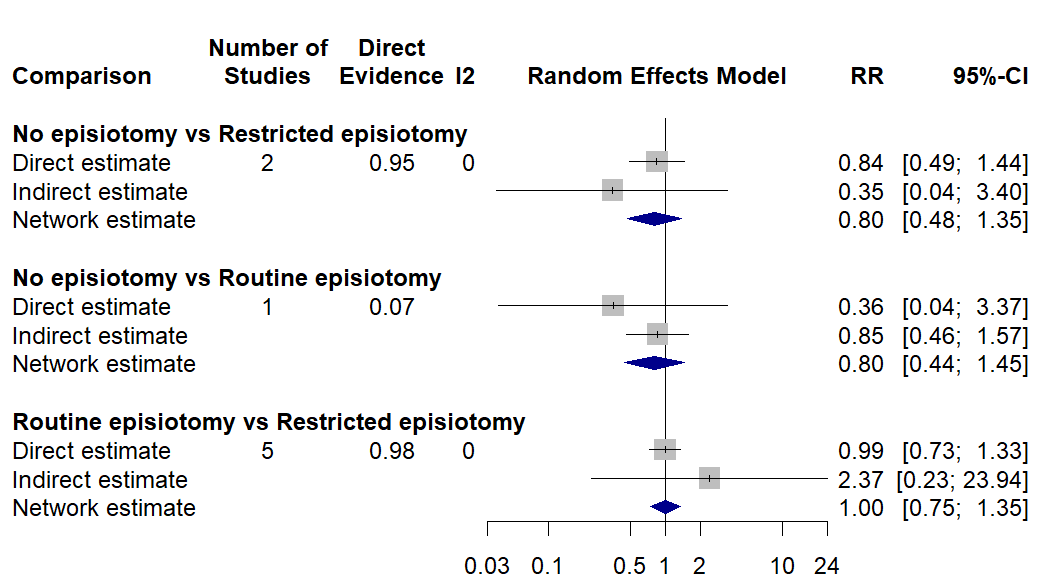
**

**Supplementary Figure S12** Pairwise meta-analysis for 1st minute Apgar score <7.

## **3.4.6.2 Network meta-analysis result and ranking** The non-episiotomy group ranked first, followed by restrictive, and finally routine episiotomy. Both non- episiotomy and routine episiotomy were comparable to restrictive episiotomy (RR = 0.80, 95% CI [0.48; 1.35]; RR = 1.00, 95% CI [0.75; 1.35]). The pooled studies were homogeneous (I² = 0%, *P* = 0.79) (Supplementary Figures S13 and S17F).


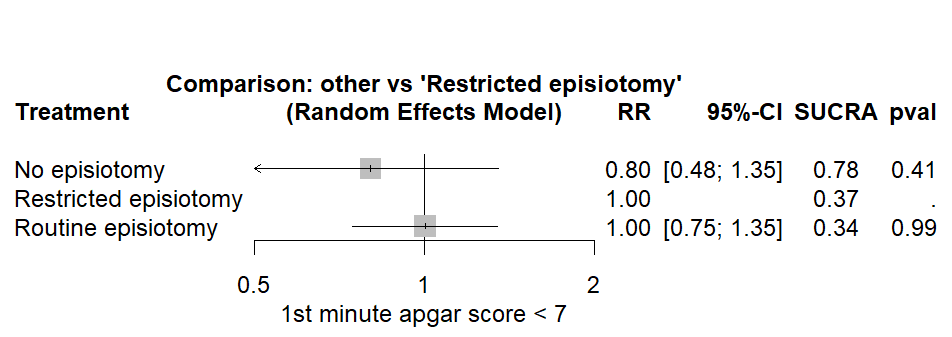


**Supplementary Figure S13** Forest plot of network meta-analysis for 1st minute Apgar score <7.

# 3.4.7 5-minute Apgar score < 7

## 3.4.7.1 Pairwise meta-analysis result

In two studies (1,3) between non-episiotomy and restrictive episiotomy, a non-significant risk reduction was observed (RR = 0.34, 95% CI [0.05; 2.25]). Similarly, in one study (10) comparing between non-episiotomy and routine episiotomy, a non-significant risk reduction was found (RR = 0.36, 95% CI [0.01; 8.64]). Additionally, one study (9) comparing between routine and restrictive episiotomies indicated a non-significant risk reduction (RR = 1.02, 95% CI [0.15; 7.10]) (**Supplementary Figures S14** and **S16G**).


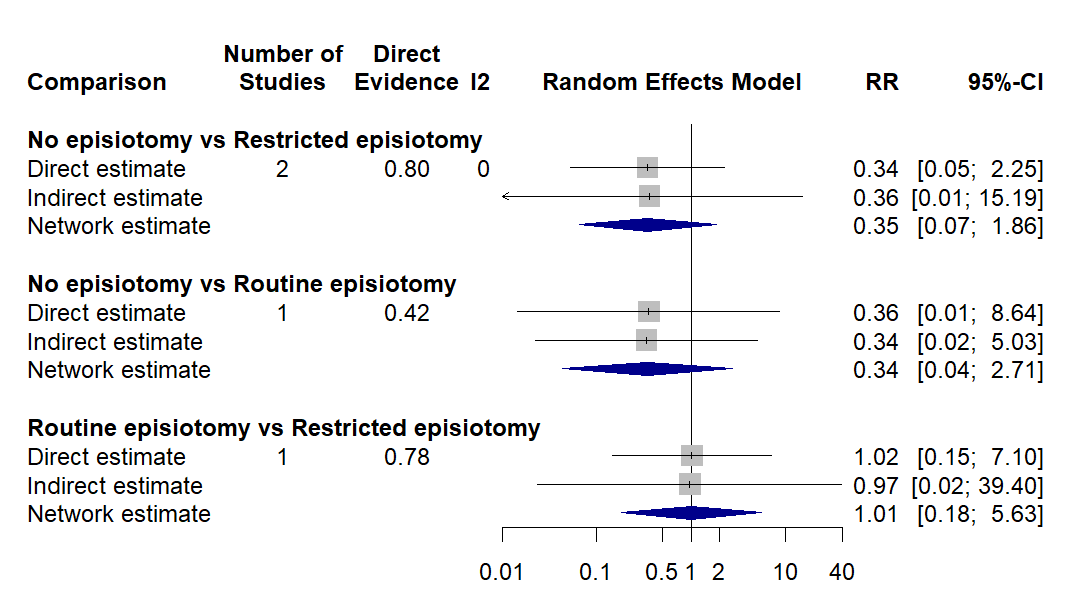


**Supplementary Figure S14** Pairwise meta-analysis for 5-minute Apgar score <7.

## 3.4.7.2 Network meta-analysis result and ranking

The non-episiotomy group ranked first, followed by the routine episiotomy, and finally the restrictive episiotomy group. The network meta-analysis revealed that both non- episiotomy and routine episiotomy were comparable to restrictive episiotomy (RR = 0.35, 95% CI [0.07; 1.86]; RR = 1.01, 95% CI [0.18; 5.63], respectively). The pooled studies were homogeneous (I² = 0%, *P* = 0.62). (**Supplementary Figures S17** and **S15G**).

**
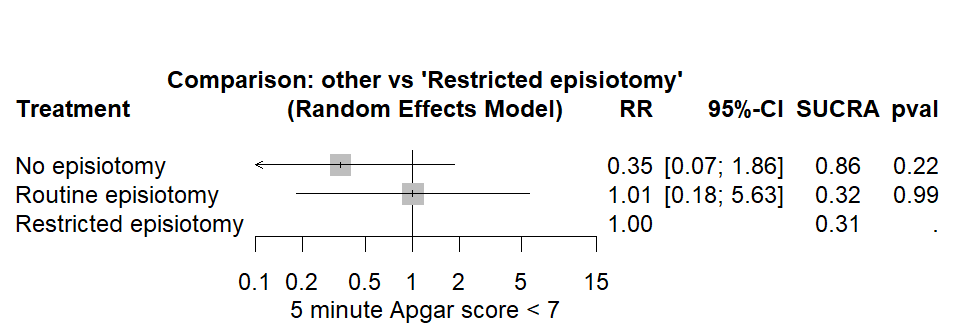
**

**Supplementary Figure S15** Forest plot of network meta-analysis for 5-minute Apgar score <7.

**
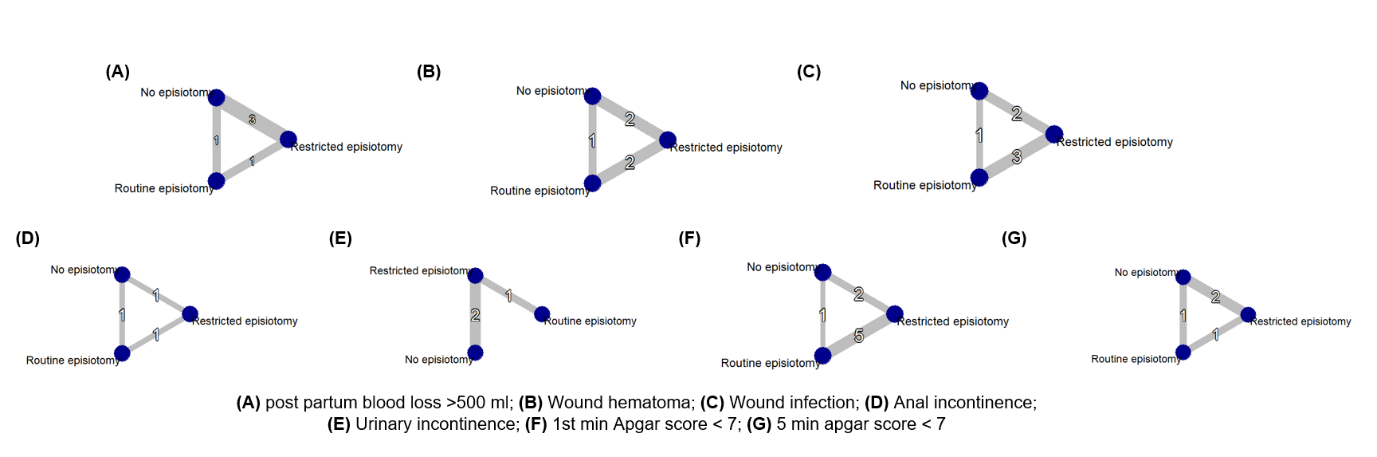
**

**Supplementary Figure S16** Network graphs for secondary outcomes.

**
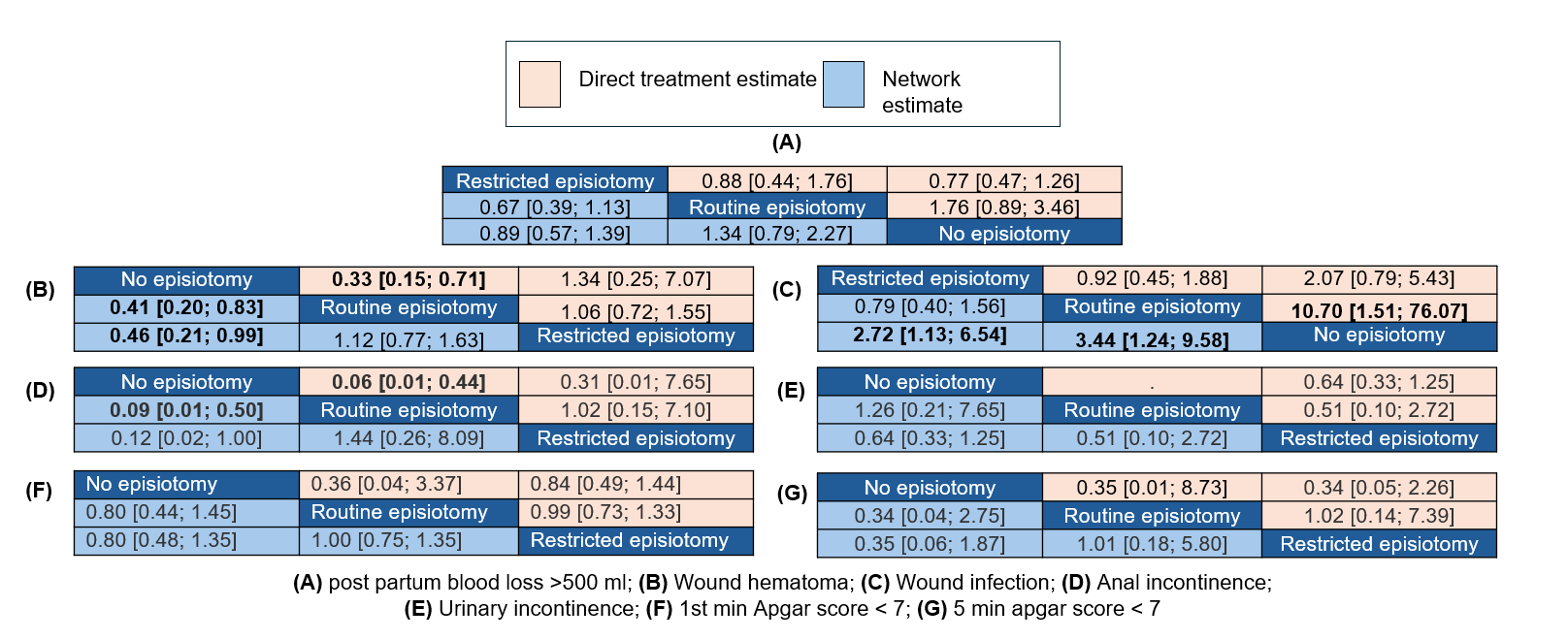
**

**Supplementary Figure S17** League tables for secondary outcomes.

**References**:

1. Amorim MM, Coutinho IC, Melo I, Katz L. Restricted episiotomy vs. implementation of a non-episiotomy protocol: A randomized clinical trial. Reprod Health. 2017 Apr 24;14(1).

2. Sagi-Dain L, Bahous R, Caspin O, Kreinin-Bleicher I, Gonen R, Sagi S. No episiotomy versus selective lateral/Mediolateral episiotomy (EPITRIAL): an interim analysis. Int Urogynecol J. 2018 Mar 1;29(3):415–23.

3. Sagi-Dain L, Kreinin-Bleicher I, Bahous R, Gur Arye N, Shema T, Eshel A, et al. Is it time to abandon episiotomy use? A randomized controlled trial (EPITRIAL). Int Urogynecol J. 2020 Nov 1;31(11):2377–85.

4. Rockner G, Henningsson A, Wahlberg V, Olund A. Eva1 uat ion of Episiotomy and Spontaneous Tears of Perineum during Childbirth. Vol. 2, Scand J Caring Sci.

5. Sangkomkamhang U, Kongwattanakul K, Kietpeerakool C, Thinkhamrop J, Wannasiri P, Khunpradit S, et al. Restrictive versus routine episiotomy among Southeast Asian term pregnancies: a multicentre randomised controlled trial. BJOG [Internet]. 2020 Feb 1 [cited 2024 Feb 4];127(3):397–403. Available from: https://pubmed.ncbi.nlm.nih.gov/31749273/

6. Ali SS, Malik M, Iqbal J, Faruqi NJ. Routine episiotomy versus Restricted episiotomy in primigravidae. Ann King Edw Med Univ [Internet]. 2004 May 18 [cited 2024 Feb 4];10(4). Available from: https://annalskemu.org/journal/index.php/annals/article/view/1272

7. Routine vs Restricted episiotomy: a randomised controlled trial. Argentine Episiotomy Trial Collaborative Group - PubMed [Internet]. [cited 2024 Apr 9]. Available from: https://pubmed.ncbi.nlm.nih.gov/7902901/

8. House MJ, Cario G, Jones MH. Episiotomy and the perineum: A random controlled trial. J Obstet Gynaecol (Lahore). 1986;7(2):107–10.

9. Murphy DJ, Macleod M, Bahl R, Goyder K, Howarth L, Strachan B. A randomised controlled trial of routine versus restrictive use of episiotomy at operative vaginal delivery: A multicentre pilot study. BJOG. 2008;115(13):1695–703.

10. Moini A, Yari REA, Eslami B. Episiotomy and third- and fourth-degree perineal tears in primiparous Iranian women. Int J Gynaecol Obstet [Internet]. 2009 [cited 2024 Feb 4];104(3):241–2. Available from: https://pubmed.ncbi.nlm.nih.gov/19159878/

11. Eltorkey MM, Al Nuaim MA, Kurdi AM, Sabagh TO, Clarke F. Episiotomy, elective or selective: A report of a random allocation trial. J Obstet Gynaecol (Lahore). 1994;14(5):317–20.

12. Sleep J, Grant A, Garcia JO. West Berkshire perineal management trial. Vol. 289, BRITISH MEDICAL JOURNAL. 1984.
